# Incorporating Noncovalent Interactions in Transfer Learning Gaussian Process Regression Models for Molecular Simulations

**DOI:** 10.1021/acs.jctc.4c00402

**Published:** 2024-07-09

**Authors:** Matthew L. Brown, Bienfait K. Isamura, Jonathan M. Skelton, Paul L. A. Popelier

**Affiliations:** Department of Chemistry, The University of Manchester, Oxford Road, Manchester M13 9PL, United Kingdom

## Abstract

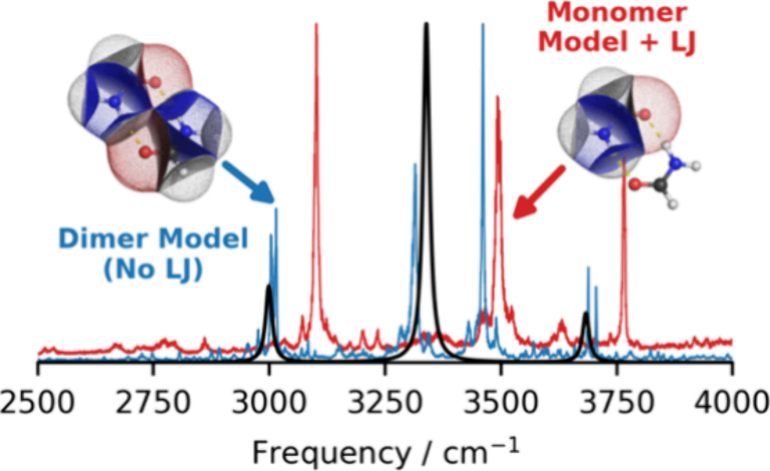

FFLUX is a quantum chemical topology-based multipolar force field that uses Gaussian process regression machine learning models to predict atomic energies and multipole moments on the fly for fast and accurate molecular dynamics simulations. These models have previously been trained on monomers, meaning that many-body effects, for example, intermolecular charge transfer, are missed in simulations. Moreover, dispersion and repulsion have been modeled using Lennard-Jones potentials, necessitating careful parametrization. In this work, we take an important step toward addressing these shortcomings and show that models trained on clusters, in this case, a dimer, can be used in FFLUX simulations by preparing and benchmarking a formamide dimer model. To mitigate the computational costs associated with training higher-dimensional models, we rely on the transfer of hyperparameters from a smaller source model to a larger target model, enabling an order of magnitude faster training than with a direct learning approach. The dimer model allows for simulations that account for two-body effects, including intermolecular polarization and charge penetration, and that do not require nonbonded potentials. We show that addressing these limitations allows for simulations that are closer to quantum mechanics than previously possible with the monomeric models.

## 1 Introduction

Noncovalent interactions play an important role in a wide range of chemical properties.^1^ In molecular crystals, these interactions contribute to polymorphism, where molecules form multiple crystal structures with different physical properties including, but not limited to, color^2^ and solubility.^3^ This makes the fundamental understanding of polymorphism an essential topic in modern structural chemistry. This is particularly the case when formulating drugs in the pharmaceutical industry, as the solubility can, for example, affect the bioavailability. Computationally, potential polymorphs can be identified through crystal structure prediction studies, where 10^3^ to 10^4^ candidate structures are generated and energetically ranked to identify low-energy polymorphs.^4^ These calculations are computationally demanding and would benefit from using force fields over dispersion-corrected periodic density functional theory, for example. However, traditional potentials are generally not considered accurate enough to capture the small energy differences between structures (typically of the order of a few kJ mol^–1^).^5^

In traditional force fields, noncovalent interactions are generally split into electrostatic and van der Waals interactions. Models for the van der Waals interactions typically take the form of a pair potential representation of dispersion and repulsion, with the Lennard-Jones^6^ and Buckingham models^7^ arguably the most well-known^8^ and implemented in several popular force field packages.^9−12^ For widely studied systems, a variety of parametrizations for these potentials are often available, although optimizing for different properties often results in different parameters, and (re)parametrizing for new systems or potential forms can be time-consuming. Moreover, our own work and that of others have shown that simulation results can be extremely sensitive to the parametrization,^13^ bringing into question their transferability and, more fundamentally, their physicality.

Machine learning (ML) offers an opportunity to model noncovalent interactions with *ab initio* quality and efficiency comparable to force fields in simulations, with the computational expense offset to the training of the models. A well-trained ML model allows for highly accurate calculations, avoiding the approximations in traditional van der Waals potentials and the difficulty of parametrizing them.

There are a number of examples of ML being successfully applied to the calculation of noncovalent interactions. This has previously been achieved for electrostatic interactions in hydrogen-bonded complexes within our own group using Gaussian process regression (GPR) models for the multipole moments up to the hexadecapole moment.^14^ In work by von Lilienfeld et al.,^15^ machine learning models provided on-the-fly predictions for environment-dependent electrostatic multipole coefficients, polarizabilities, and decay rates of valence atomic densities. The predicted properties were then used with physics-based potentials to enable accurate calculation of intermolecular energy contributions including electrostatics, charge penetration, repulsion, polarization, and many-body dispersion. The ML force field CLIFF^16^ similarly combines physics-based equations with ML, utilizing a symmetry-adapted perturbation theory (SAPT) energy decomposition scheme to define advanced functional forms and ML models to automate the parametrization of the potentials. Finally, many-body interactions have been incorporated into Gaussian process regression (GPR) models using the electron deformation density interaction energy machine learning (EDDIE-ML) algorithm,^17^ which predicts interaction energies as a function of the Hartree–Fock electron deformation density. While limited to dimers in its initial version, EDDIE-ML has recently been extended to account for three-body interactions.^18^ All three models have been able to capture many-body interactions with sub-kJ mol^–1^ accuracy in single point calculations but have yet to be used in molecular dynamics (MD) simulations.

An issue with high-accuracy ML models is that training can be time-consuming. This situation can be improved through the selection of appropriate algorithms, in particular, those that limit the number of *ab initio* calculations or the computational cost required to prepare the training set. An example is transfer learning (TL), which uses knowledge from a “source” task to bias and therefore improves the learning on a related “target” task.^19^ For the construction of ML potentials, a source model can be trained on a large data set of low-level *ab initio* calculations, and the accumulated knowledge is used to readjust a target model with fewer expensive higher-level calculations.^20^ This prevalent TL workflow is often exploited to reduce the risk of overfitting artificial neural networks on small data sets but is not regularly used when preparing kernel-based models such as GPR models.

FFLUX^21,22^ is a next-generation force field that utilizes GPR models trained on atomic energies and multipole moments from the interacting quantum atom^23^ (IQA) energy partitioning scheme. These models allow for flexible molecules with geometry-dependent multipole moments up to the hexadecapole. To our knowledge, FFLUX is the only force field with geometry-dependent multipole moments, with the AMOEBA+CF force field, for example, having only geometry-dependent charges.^24^ The FFLUX force field is implemented in the DL_FFLUX package and can be used for a wide range of simulations including on gas phase clusters,^25^ liquids,^26^ and molecular crystals.^27^

Previously, FFLUX has been used with monomeric models, meaning that the GPR models have been trained on monomers of molecules of interest. Monomeric models can accurately describe short-range (intramolecular) polarization. Long-range (intermolecular) interactions are described using the predicted multipole moments through a smooth particle mesh Ewald (SPME) summation,^28,29^ but there is no explicit long-range polarization or charge penetration as in the schemes discussed above. Furthermore, as the GPR model of a monomer does not have “knowledge” of intermolecular interactions, van der Waals interactions are described using a Lennard-Jones potential. Despite these limitations, monomeric models have successfully been used in simulations of liquid water^26^ and formamide crystals.^27^ In the latter study, phonon calculations within the harmonic approximation produced a reasonable representation of the phonon density of states obtained from periodic dispersion-corrected DFT calculations, and calculated Helmholtz free energies recovered the expected ranking of the known α and β polymorphs. While this work demonstrated the potential of FFLUX for calculations of solid-state polymorphism, it also exposed limitations of the Lennard-Jones parametrization of the nonbonded interactions.

Intermolecular interactions can be accounted for within the FFLUX methodology by training models on oligomeric or *N*-meric clusters. In this work, we provide proof-of-concept results showing that GPR models of clusters incorporating non-electrostatic intermolecular interactions can be prepared and used in FFLUX simulations. Following our previous work on formamide,^25,27^ we select the formamide dimer as a test case. Training on clusters increases the dimensionality of the system, slowing down the training process, but we mitigate this using a new implementation of transfer learning in our in-house machine learning engine FEREBUS.^30−32^ The dimer models are employed in MD-based optimizations, single point calculations, and finite temperature MD simulations to calculate vibrational frequencies and simulate infrared (IR) spectra. Comparison of results from our previous monomeric model and the new dimer model demonstrates that the latter produces results closer to the quantum mechanical method used for training. We note that, at the time of writing, the dimer model cannot be applied to structures larger than a dimer, as to do so requires significant changes to the implementation of FFLUX. However, these results highlight the clear benefits of doing so and provide a pathway to force fields that can accurately describe a range of intermolecular interactions without the need for nonbonded potentials.

## 2 Methods

### 2.1 Quantum Chemical Topology

**Figure 1:**
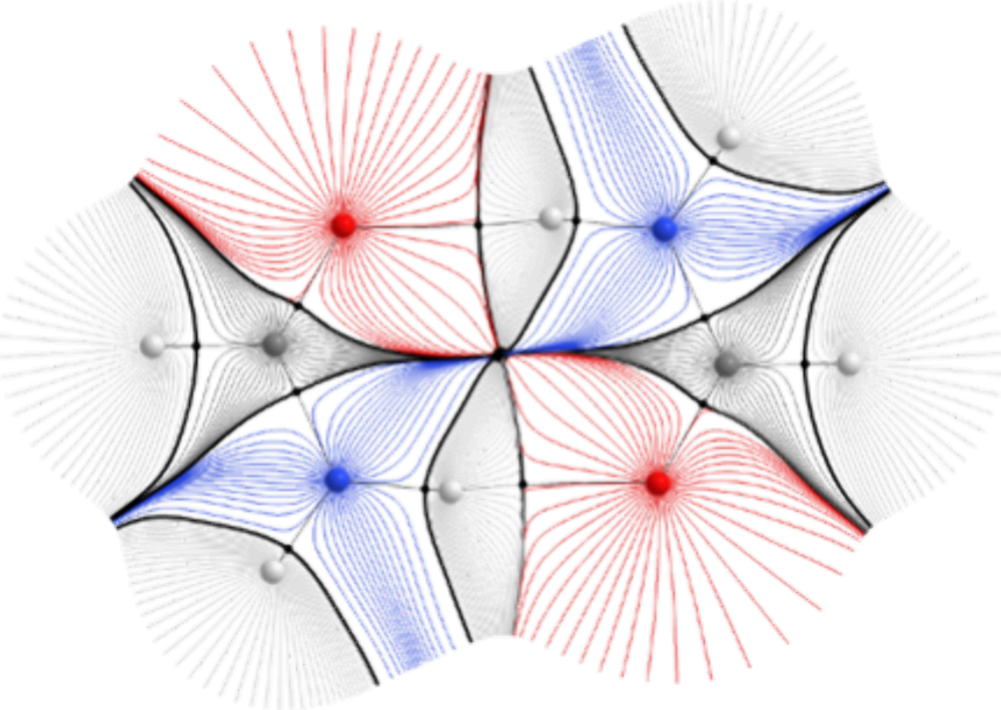
Partitioned formamide dimer with gradient paths shown as thin lines. Atom colors: H, light gray; C, dark gray; N, blue; O, red. Thick black lines show interatomic (zero-flux) surfaces that terminate at one of two types of saddle point marked by tiny black disks. The first is termed a bond critical point and occurs between atoms. The second is named a ring critical point and is found at the center of the doubly hydrogen-bonded eight-membered ring formed by the formamide dimer.

Quantum chemical topology (QCT) encapsulates a group of methods that share the idea of a vector field partitioning a quantum mechanical function. In the construction of GPR models for FFLUX, two QCT methods are important. The first method is the quantum theory of atoms in molecules (QTAIM),^33^ where a gradient vector field is applied to the electron density to reveal a series of trajectories termed gradient paths (highlighted in Figure 1). Gradient paths can be seen as trajectories of infinitely short gradient vectors, updated at each point in space, that ascend toward, and terminate at, critical points in the electron density.

A collection of gradient paths makes up an object called a topological atom, which is bounded by a surface of zero-flux (i.e., an interatomic surface, IAS). These surfaces are a collection of gradient paths obeying eq 1,
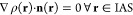
1where ρ is the electron density, and **n**(**r**) is a normal vector to the surface at the point **r**. These zero-flux surfaces allow for the partitioning of a molecular electron density into its constituent topological atoms without the need for a reference electron density.

The second QCT method important to FFLUX is the IQA partitioning, which extends QTAIM to be independent of the atomic virial theorem and allows for nonstationary geometries to be partitioned into chemically meaningful interactions. The virial theorem in QTAIM links the kinetic and potential energies of atoms in a system at stationary points. However, by calculating the potential energy from scratch, IQA partitions the one- and two-particle density matrices into energetic terms that, when summed, recover the total wave function energy for any molecular geometry. IQA is a general and rigorous scheme that has been applied to a wide range of systems of different chemistries and sizes.^34−38^

An atomic IQA energy, , can be broken down into intra- () and interatomic () contributions as shown in eq 2,
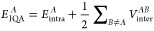
2

Both terms can be further decomposed into various kinetic and potential energies *T* and *V*:
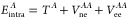
3
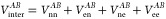
4

The subscripts in eqs 3 and 4 indicate nuclear (n) and electronic (e) interactions within or between the superscript topological atoms *A* and *B*.  can be further partitioned into Coulomb and exchange-correlation energies, allowing the purely electrostatic (classical) terms to be grouped together as , and thus,  can be written as
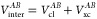
5where  is the exchange-correlation energy.

### 2.2 Gaussian Process Regression

#### 2.2.1 Direct Learning

This subsection explains key details of the way we used to train GPR models and still do, which we now call direct learning to distinguish it from transfer learning described in the next subsection. The atomic energies from the IQA partitioning and the multipole moments appearing in the Laplace expansion of the *V*_cl_ terms corresponding to a series of molecular geometries make up the training data for the GPR models used in FFLUX simulations. GPR, also known as kriging, is a supervised machine learning method where each model is defined by a training set and a set of hyperparameters. A GPR model consists of a set of *n* training points (**X**, **y**) where **X** is a set of *D*-dimensional input vectors containing *D* features, and **y** is a vector of corresponding outputs (IQA energies and multipole moments in this case).

The input vectors in our GPR models are molecular geometries expressed in an atomic local frame (ALF). Each atom, *A*, has its own unique ALF. The atom *A* defines the origin. Two atoms, identified as the highest and second-highest priority atoms by the Cahn–Ingold–Prelog rules and denoted *A*_*x*_ and *A*_*xy*_, respectively, are used to define the *x*-axis and *xy*-plane. The *z*-axis is then constructed orthogonally to form a right-handed axis system. The first three features of each model are then the *A*–*A*_*x*_ and *A–A*_*xy*_ distances and the *A*_*x*_–*A–A*_*xy*_ angle. Any remaining atoms in the system are described in spherical coordinates relative to the ALF. Each model therefore has 3*N* – 6 features, where *N* is the number of atoms being trained for.

A covariance function, or kernel, *k*(**x**, **x**′), must be chosen that captures the similarity between every pair of points **x** and **x**′. The kernel used in this work is a modified radial basis function (RBF) kernel that accounts for every third feature being an angular feature ranging from −π to π in value. This kernel, named the RBF-Cyclic kernel, is shown in eq 6,
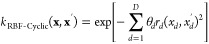
6
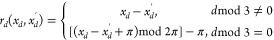
where the hyperparameters θ_*d*_ scale the distance between the *D* features of the training points **x** and **x**′. Training a GPR model entails finding an optimal set of θ, which is generally achieved by maximizing the marginal log-likelihood function (or its concentrated equivalent) using metaheuristic algorithms. In this task, during each iteration, the covariance matrix, **R**, must be constructed and inverted:
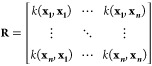
7

This inversion is typically carried out by Cholesky decomposition. Gaussian noise can also be added along the diagonal to improve the numerical stability of operations involving **R**. This approach to training GPR models is commonly referred to as type II maximum likelihood (ML-II) and is the default protocol in most GPR packages. However, the ML-II approach can suffer from the propagation of numerical errors^39^ and can be very sensitive to outliers^40^ or non-Gaussian noise.^41^ Here, we use the iterative hold-out cross-validation (IHOCV) protocol described in our previous work.^39^ In the IHOCV protocol, the predictive root-mean square error (RMSE) over a fixed and representative internal validation set serves as the cost function to be minimized,
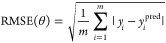
8where *m* is the number of points in an internal validation set and *y*_*i*_ and  are the true and predicted outputs for the *i*-th validation point, respectively. At each iteration of the IHOCV protocol, our metaheuristic optimizer updates the list of candidate solutions based on a well-defined search mechanism. Each solution **θ**^*g*^, where the superscript *g* runs over the total number of possible solutions, is a vector of 3*N* – 4 hyperparameters including 3*N* – 6 feature-scaling hyperparameters (θ_*d*_) plus the regularization noise added to the diagonal of the covariance matrix and a kernel prefactor, which is here fixed at 1 for compatibility with DL_FFLUX. Once this solution is generated, it is used to build a temporary (“intermediary”) model. The quality of this model is determined by the cost function in eq 8. Finally, solutions are ranked and the process is repeated until the maximum number of iterations is reached. Upon completion, the best solution among all the candidates is retained and used to build the optimized model.

Once a model has been trained, predictions are made using
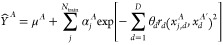
9where  is the predicted value for a property *A*, μ^*A*^ is the average output value across all the training points, and  is the weight of the *j*th training point.

Training of models is achieved using our in-house machine learning engine FEREBUS,^30−32^ which is written in the Fortran90 programming language. FEREBUS has recently been updated to include on-the-fly validation of models and several metaheuristic optimizers, and a “light” version featuring the best performing optimizer and kernel, as found by Isamura and Popelier in ref (32), has also been created. This FEREBUS-LIGHT version was used to construct the direct and transfer-learned models in the present work.

#### 2.2.2 Transfer Learning

Transfer learning uses information from a source model to train a target model. In the case of the GPR models trained here, the source model denoted *S* is trained on a subset of the training data to estimate hyperparameters for the target model, *T*, trained on the whole training set. The fraction of the training set used in the source model is specified by the knowledge compression coefficient, η,
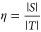
10where the vertical bars denote the size of the set they are surrounding.

Taking an example, for a 1000-point target model, a 10-point source model corresponds to η = 0.01, in which case 1% of the 1000 training geometries are chosen to obtain an initial set of hyperparameters for training the target model. In FEREBUS-LIGHT, these geometries can be selected *via* random or passive sampling. During the overall training process the hyperparameters of the source and target model are relaxed according to a ratio ζ called the relaxation weight, which is defined by
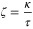
11where τ is the maximum total number of iterations for optimizing both the source and models and κ is the number of relaxation steps used to optimize the target model only. Again, taking an example, if τ = 1000 steps and ζ = 0.1, then 100 relaxation steps are used for optimizing the target model hyperparameters and the remaining 900 steps for optimizing the source model hyperparameters. A special case of the transfer learning, as implemented here, is when ζ = 0, where the hyperparameters from the source model are used for the target model without further relaxation. This extreme case is termed “frozen-seed” transfer learning. A systematic study demonstrating the performance of this protocol for a range of systems is currently underway, and the results will be reported in a future publication.

## 3 Computational Details

### 3.1 Preparation of GPR Models

In FFLUX simulations, each atom in a system (where “system” refers to the molecule or oligomer being trained for) requires a GPR model for its atomic energy and for each component of its multipole moments up to the hexadecapole moment. The latter comprise 25 components across all multipole moments in the spherical tensor form, which is more compact than the Cartesian form. Each atom therefore has 26 GPR models associated with it in total, enabling the description of both its short- and long-range energies. The models generated here were constructed using our in-house codes ICHOR^42^ and FEREBUS-LIGHT. ICHOR is a Python package that pipelines the programs required to generate the training data for models (AMBER18,^12^ CP2K,^43^ GAUSSIAN,^44^ and AIMAll^45^), while FEREBUS-LIGHT is the GPR engine used to train models.

#### 3.1.1 Data Set Generation

For formamide monomer models, a data set of geometries was generated from a 1 ns AMBER simulation at 300 K using the GAFF2 force field. Using a time step of 1 fs, 10^6^ points (i.e., data points or molecular geometries) were generated and then reduced to 15,000 points by sampling evenly spaced points throughout the trajectory. For each of these 15,000 points, wave functions were calculated using the *ab initio* program GAUSSIAN16 at the B3LYP/6-31+G(d,p) level of theory. Atomic energies and multipole moments were then obtained using the IQA partitioning implemented in AIMAll. This process was performed using the ICHOR pipeline.

**Figure 2:**
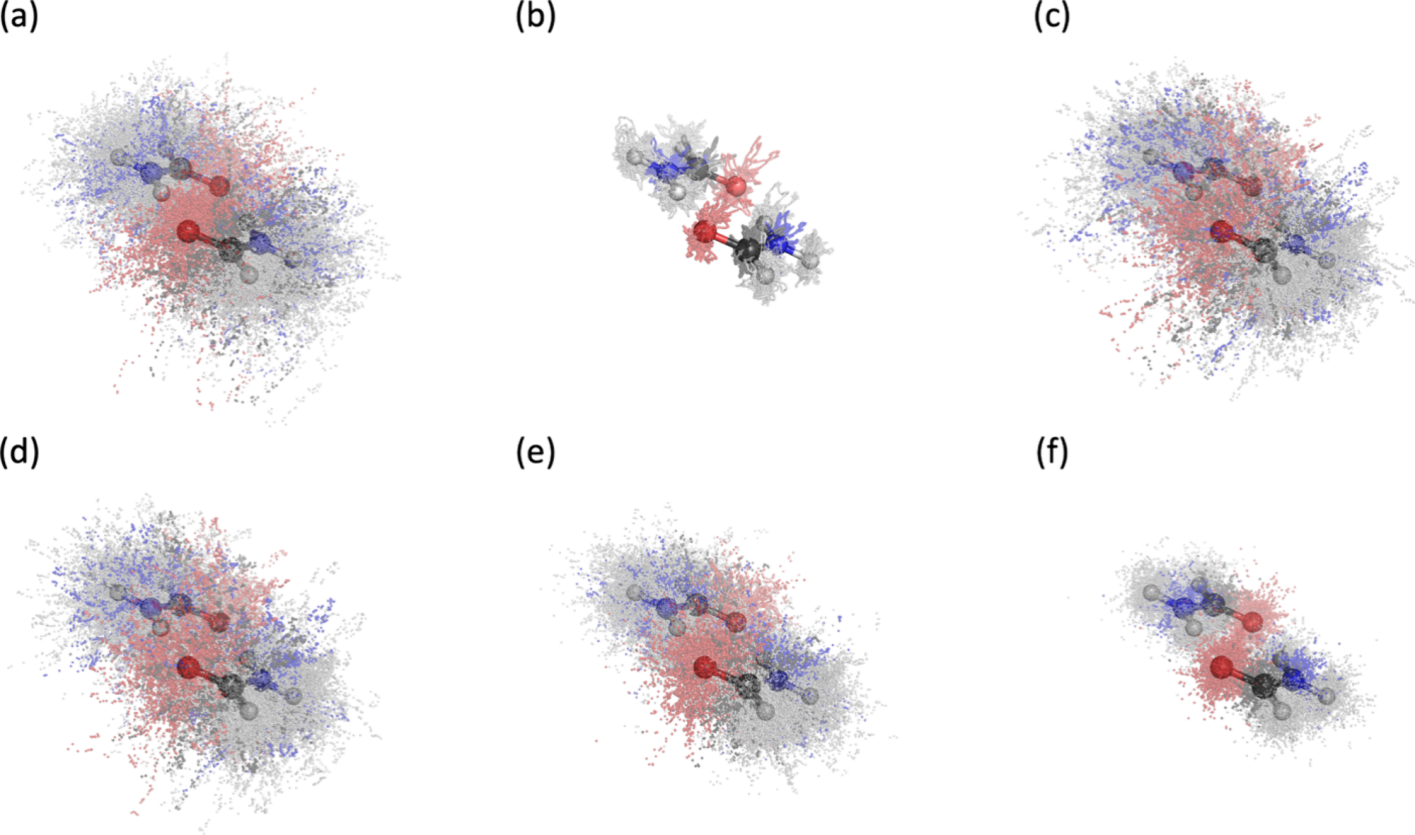
Mist plots showing the trajectories of a formamide dimer simulation from (a) an AMBER simulation at 300 K and (b) a CP2K simulation at 300 K. Panels (c) to (f) show a sample of 15,000 points from the hybrid trajectories created from a combination of the trajectories in panels (a) and (b) using root-mean square deviation thresholds of (c) 1 Å, (d) 0.7 Å, (e) 0.4 Å, and (f) 0.2 Å to control the geometry selection.

Similarly, a 70 ps AMBER simulation of a formamide dimer was initially used to generate dimer geometries, but the large variation in geometries produced a domain space that would have been difficult to accurately capture in a comparable number of points to the monomer model (see mist plot in Figure 2a). Models of oligomers require more points to be modeled with similar accuracy to their monomeric counterparts. This is because the increased number of training points increases the dimensions of the covariance matrix, which must be inverted at each iteration of the training. This is achieved with the Cholesky decomposition, which scales as  where *n* is the number of training points. In the future, we plan to substitute the standard Cholesky decomposition used during the training with a GPU-enabled iterative solver with better scaling for the inversion of the covariance matrix.^46^

To limit the domain space to a more manageable size, geometries from a 10 ps CP2K simulation of the formamide dimer (B3LYP/6-31G* at 300 K using a Nosé–Hoover thermostat with a relaxation time of 50 fs and a time step of 1 fs) were used as a “control”. The idea here is that the domain space of the CP2K trajectory will be a smaller subset of the geometries covered in the AMBER trajectory, and geometries in the latter can then be excluded based on a root-mean square deviation (RMSD) threshold. Each geometry in the 10,000 point CP2K trajectory was compared to each of those in the AMBER trajectory, and AMBER geometries below the threshold were selected to form a “hybrid” trajectory in combination with all of the CP2K points. As the threshold is decreased, the domain space of the hybrid trajectories is reduced as shown in Figure 2c–f. This reduction simplifies the task for machine learning, meaning that fewer points are required for an accurate model. For the dimer models in this work, a hybrid data set of approximately 60,000 points was generated using a threshold of 0.4 Å and a random sample of 15,000 geometries was selected from this data set and treated in the same way as the monomer geometries. Figures S1.1–S1.5 of Section 1 of the Supporting Information show the distributions of the energies and atomic charges in each data set.

The two data sets were then filtered by the recovery error, *E*_recov_, which is the difference between the wave function energy, *E*_wfn_, and the sum of the atomic energies of all the atoms in the system (i.e., the IQA total energy of the system):
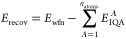
12Geometries with a recovery error greater than 1 kJ mol^–1^ were excluded from the training data sets, leaving 14,999 monomer geometries and 14,910 dimer geometries to be sampled.

#### 3.1.2 Sampling

Uncertainty-enhanced stratified sampling (UESS) was employed to generate a training set of 5000 points and internal and external validation sets of 750 and 1500 points, respectively, from the data sets, for both the dimer and monomer models. The internal validation set is used during the optimization of hyperparameters within the IHOCV protocol, while the external validation is used as a test set for the models. UESS acts as a combination of stratified and passive sampling.^47^ The data set is first split into subpopulations covering the range of the target properties. Within each subpopulation, the most diverse geometries are then selected (i.e., the geometries that are most different from each other in the feature space). This method improves upon (standard) stratified random sampling by ensuring that the training and validation sets are suitably representative of the whole data set and capture the diversity of each subpopulation from the stratified sampling.

#### 3.1.3 Training

In this work, a series of models for the formamide monomer and dimer were trained using the newly implemented transfer learning in FEREBUS-LIGHT and compared to models constructed using direct learning. In the following, “direct models” refer to the models trained directly on the training sets with no transfer of hyperparameters from smaller source models.

To investigate the cost-accuracy benefit of using transfer learning, we tested knowledge compression coefficients (η) of 0.001, 0.01, and 0.1, respectively, using 5, 50, and 500 points to construct source models for 5000-point target models and relaxation weights ζ of 0, 0.001, 0.005, 0.01, 0.05, 0.1, and 0.2, with 1000 iterations in total. This combination of 3 η values and 7 ζ values leads to 3 × 7 = 21 possible settings, all of which were investigated. The models trained in this paper used a random sample to generate the source models for TL, but a series of models were prepared using passive sampling for comparison. These models are discussed in Section 2 of the Supporting Information, and a comparison to the transfer-learned models obtained using random sampling is given in Tables S2.1 and S2.2.

Optimization of hyperparameters was performed using an enhanced gray wolf optimizer (GWO-RUHL)^32^ and the IHOCV training protocol.^39^ The GWO-RUHL method was identified as the best performing of the series of metaheuristic optimizers tested in ref (32), and the IHOCV protocol produces more consistent models as described in Section 2.2. The level of noise was optimized for each model with upper and lower bounds of 10^–4^ and 10^–10^, respectively.

### 3.2 Molecular Simulations

Models were initially tested for geometry optimizations in FFLUX. For this purpose, the optimized monomer and dimer geometries at the B3LYP/6-31+G(d,p) training level of theory were distorted by 15% along each of the 3*N* – 6 normal mode coordinates to generate a set of 12 and 30 input geometries. As DL_FFLUX is built on DL_POLY 4, many of the subroutines available in DL_POLY can be used in FFLUX simulations. MD simulations were performed in the *NVT* ensemble with a 1 fs time step and a Nosé–Hoover thermostat with a 0.2 ps relaxation time. Optimizations were run using the DL_POLY “Zero Kelvin” optimizer, in which atoms move in the direction of the calculated forces and torques but are not allowed to gain a velocity greater than they would at 10 K, thereby forcing the geometry into the nearest local minimum. These optimizations were run for 5 ps. This MD-based optimizer was chosen instead of the gradient-based methods implemented in DL_POLY as we have found in previous work that this produces energies more consistent with the training level of theory.^48^ Finite temperature MD simulations were also performed with the same parameters but at finite temperature (i.e., with the “Zero Kelvin” directive removed). Calculations on formamide dimers using the monomeric models required a set of nonbonded parameters, and a description of how these were derived is given in Section 4.2.1.

## 4 Results and Discussion

### 4.1 Transfer versus Direct Learning

#### 4.1.1 Model Training

The aim of the transfer learning implemented in FEREBUS is to speed up the training of the GPR models while maintaining the accuracy of direct learning. To test this, a series of 5000-point TL models were generated using different knowledge compression coefficients and relaxation weights as described in Section 3.1.3.

**Figure 3:**
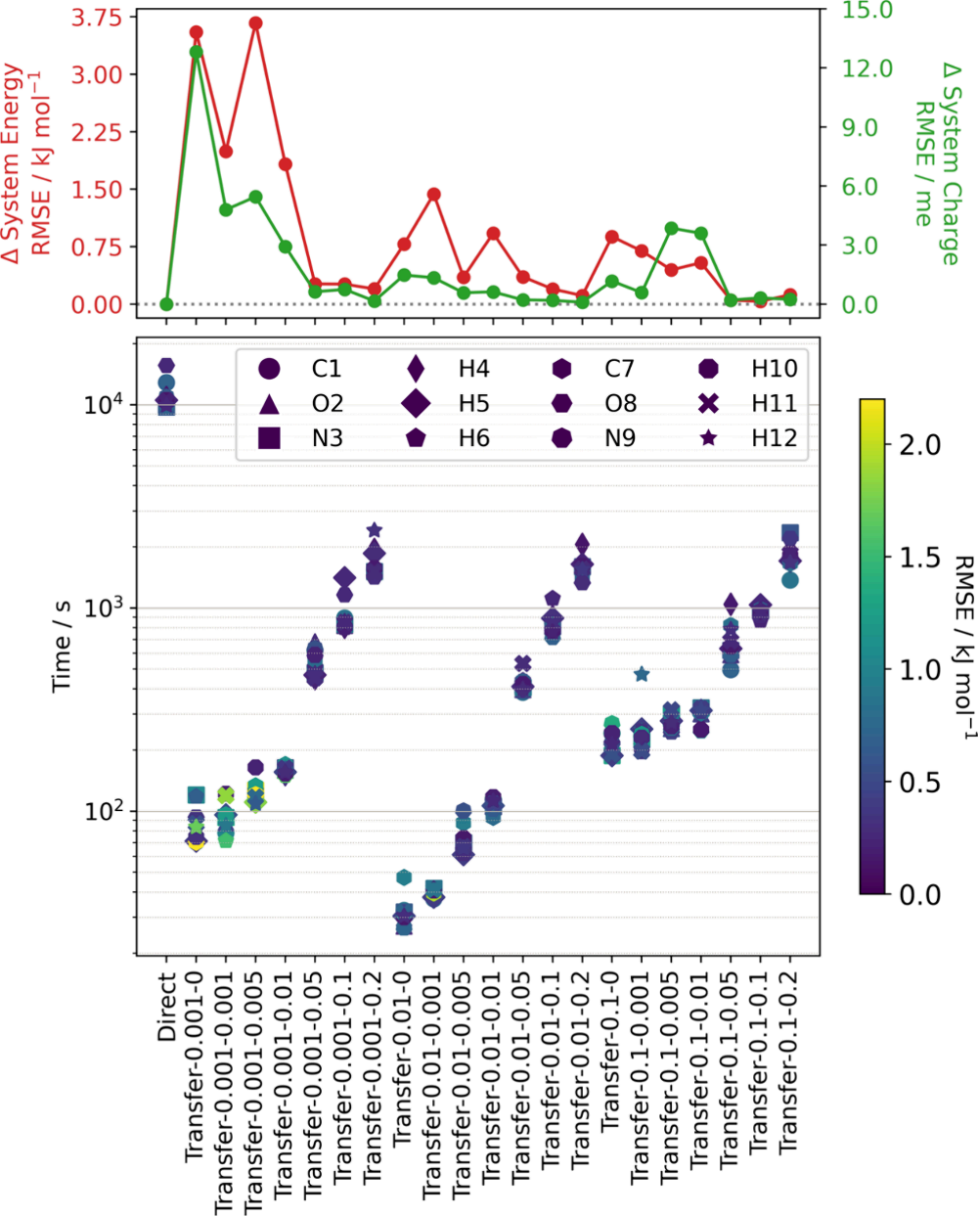
(Top) RMSE in the energies (red) and total system charges (green) of formamide dimers in a 1500-point external validation set predicted by transfer-learned models, compared to a direct learning model. (Bottom) Training times for individual atoms on 20 cores of a single compute node comprising two Intel “Cascade Lake” Xeon Gold 6230 chips compared to the RMSEs in the predicted atomic energies. The parameters for the various models are indicated by labels of the form “Transfer-η–ζ”, where η are the knowledge compression coefficients and ζ are the relaxation weights that cover the 3 × 7 = 21 possibilities outlined in the main text.

To choose which TL model to take forward for production calculations, the training times were compared with how well the model reproduced the atomic energies and the total energies and charges of the geometries in the external validation set, captured by the root-mean-square error (RMSE). The results for the dimer model are shown in Figure 3. We note that the overall charge should be zero for the neutral dimer; deviations from neutrality come from the training data, where integration errors during the IQA partitioning may result in a nonzero total charge.

The training time for transfer-learned models can be over 2 orders of magnitude faster than for a direct-learned model, but there is generally a cost/accuracy trade-off whereby they tend to show larger errors in predicted properties. However, it is still possible to achieve an order of magnitude faster training without significant loss of accuracy. A similar comparison for the formamide monomer is given in Section 3 of the Supporting Information (Figure S3.1) and similarly shows that transfer learning offers a significant speed-up in training time with little impact on the accuracy of the resulting GPR model.

In the model with η = 0.1 and ζ = 0.1, the RMSEs of the atomic energies are all below 0.5 kJ mol^–1^ and the RMSE in the total system energy and charge are only 0.04 kJ mol^–1^ and 0.3 milli-electron (me), respectively, relative to the direct model, but the training was an order of magnitude faster. This model was therefore selected for further calculations.

**Figure 4:**
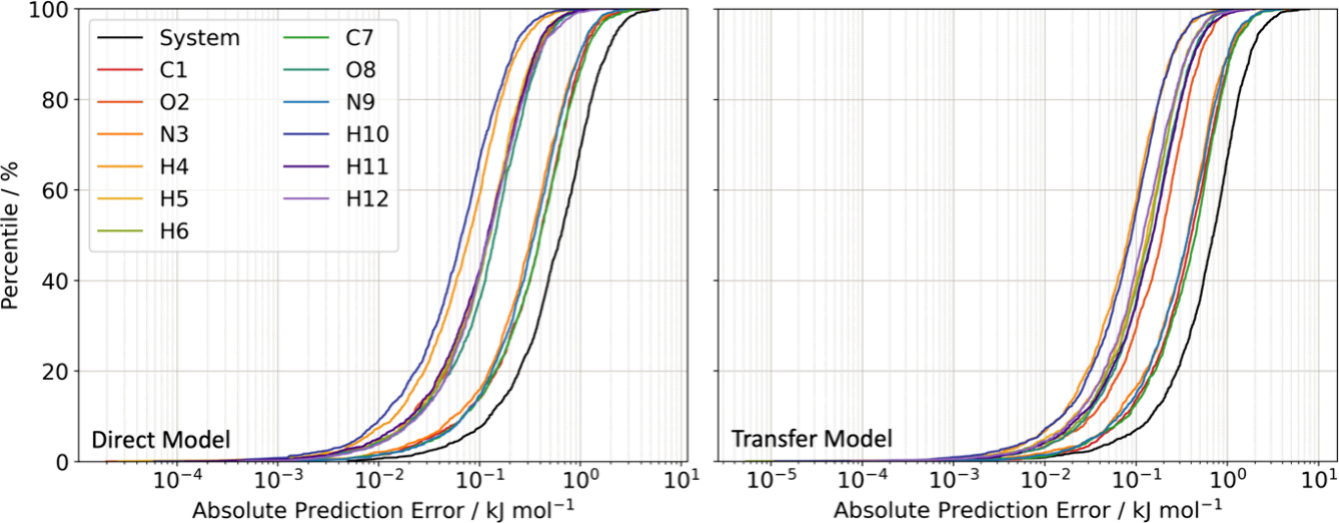
S-curves showing absolute prediction errors in the IQA atomic energies from the direct- and transfer-learned formamide dimer models compared in this work. Error distributions for the atomic energy errors are shown with colored lines, while the distribution of errors in the total system energies (i.e., the error in the energies of the whole dimers) is shown by the black line.

An additional way to assess the accuracy of a model is through the cumulative error distributions across the external validation set, termed S-curves. For each point in the test set, absolute prediction errors, PE, are calculated by
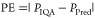
13where *P*_Pred_ is a predicted property, and *P*_IQA_ is the “true” value from the IQA decomposition. The prediction errors are then arranged from the smallest to largest and plotted as a cumulative percentile. The S-curves for the direct- and transfer-learned dimer models are compared in Figure 4.

This comparison shows that the two models perform similarly across the test set, with mean absolute errors (MAEs) of 0.88 and 0.85 kJ mol^–1^ for the transfer- and direct-learned models, respectively, and RMSEs of 1.19 and 1.15 kJ mol^–1^. The transfer-learned model also has a slightly higher maximum error of 7.9 kJ mol^–1^, which is 1.7 kJ mol^–1^ greater than the direct model. Given the significant speed-up in training, the differences between the two models are sufficiently small that we conclude that transfer learning, with appropriate parameters, can produce predictions of a similar quality to a directly learned model but with a substantial reduction in training effort. A similar comparison was performed for the direct- and transfer-learned monomer models trained for this paper, and the S-curves are compared in Section 3 of the Supporting Information (Figure S3.2). As the monomer model requires the use of the multipole moment models for dimer simulations, S-curves for each component of the dipole, quadrupole, octupole, and hexadecapole moments are also shown in Figures S3.3–S3.27.

#### 4.1.2 Geometry Optimizations

GAUSSIAN16 was used to optimize and perform a frequency calculation on the formamide monomer and dimer at the B3LYP/6-31+G(d,p) training level of theory. The normal mode coordinates were then used to generate 12 and 30 distorted geometries by applying a 15% distortion along each of the 3*N* – 6 normal modes. From B3LYP/6-31+G(d,p) single point energy calculations, the distorted geometries differ by at most 70 kJ mol^–1^ from the equilibrium geometries, therefore presenting a reasonable challenge for geometry optimizations using the models.

The distorted geometries were optimized in DL_FFLUX using the “Zero Kelvin” molecular dynamics optimizer as outlined in Section 3.2. The final geometry of a 5000-step trajectory was taken as the optimized geometry. In all cases, the absolute energy difference between the configurations in the final and penultimate steps was less than 10^–4^ kJ mol^–1^ and the optimizations were therefore considered converged.

The geometries and energies of the FFLUX-optimized monomers and dimers are compared to the corresponding B3LYP/6-31+G(d,p)-optimized systems in Table 1.

**Table 1:** Average Energy, *E*_Avg_, of the Optimized Geometries from a Set of 12 Distorted Monomer and 30 Distorted Dimer Configurations^a^

**model**	***E*_Avg_/kJ mol^–1^**	**(***E*_B3LYP_*– E*_Avg_**)**/**kJ mol^–1^**	****σ**_*E*_**/kJ mol**^**–1**^**	****RMSD**_Avg_**/Å****	****σ**_RMSD_**/Å****
monomer direct	–446,100.7	–0.07	3.0 × 10^–5^	0.002	1.3 × 10^–4^
monomer transfer	–446,100.8	0.00	3.3 × 10^–5^	0.001	1.6 × 10^–4^
dimer direct	–892,257.5	–0.99	4.6 × 10^–5^	0.01	2.3 × 10^–4^
dimer transfer	–892,257.2	–1.32	4.0 × 10^–5^	0.02	2.5 × 10^–4^

a The difference between *E*_Avg_ and the energy at the training B3LYP/6-31+G(d,p) level of theory (*E*_B3LYP_ – *E*_Avg_), and the average RMSD between the optimized and reference structures are also given, together with the respective standard deviations.

The energy and geometry of the formamide monomer are captured very well by both the direct- and transfer-learned models. The transfer-learned model in fact obtains a better (average) representation of the monomer than the direct-learned model, but the difference between the two models is small enough to consider them consistent with each other. Comparison of the energies to the reference B3LYP/6-31+G(d,p) values shows that the GPR models used in FFLUX are capable of sub-kJ mol^–1^ accuracy, as has been shown in several previous studies.^25,26,49−51^ This accuracy in principle makes the models useful for studying a wide range of systems and problems—a prime example is polymorphism in molecular solids, given that polymorphs typically differ in energy by only a few kJ mol^–1^.^5^

The two dimer models are less successful at capturing the geometry and energy obtained from the training level of theory, but this is understandable given that the higher dimensionality of the dimer makes it more challenging to model. Nevertheless, the energy predictions from both models are well within the threshold of “chemical accuracy” (approximately 4.2 kJ mol^–1^), and the direct- and transfer-learned models differ from each other by less than 1 kJ mol^–1^. Given the order of magnitude speed-up in training time, we consider this to be satisfactory.

It should be noted that, due to the stochastic procedure for selecting training points for the source model in FEREBUS, it is possible that another transfer-learned model with the same η and ζ would perform differently. This is particularly a problem with frozen-seed models, where hyperparameters from the source model are not optimized. One way to avoid this ambiguity, and to ensure that training a model with a given set of η and ζ produces consistent results, would be to use source models trained from points that are consistently selected from the data set. This can be achieved using the passive sampling implemented in FEREBUS. Section 2 of the Supporting Information discusses transfer-learned models prepared using passive sampling to select points for the source model, with a series of models tested in Tables S2.1 and S2.2. We find that using passive sampling for generating source models generally produces better consistency and has a larger impact when smaller source models are used. For example, the standard deviation in the MAE for a series of carbon atom models with η = 0.01 was 0.132 kJ mol^–1^ with a randomly sampled source model but was reduced to 0.005 kJ mol^–1^ with passive sampling. Because this is a proof-of-concept study, and since passive sampling incurs a slightly larger computational cost, we proceeded with the transfer-learned model generated using random sampling, but passive sampling of source models will be tested more thoroughly in a future study.

#### 4.1.3 Vibrational Frequencies and Infrared (IR) Spectra

An alternative assessment for how well the models describe the potential energy surface is the sensitive test of predicting vibrational frequencies. To carry this out, we used the finite-difference method implemented in the Phonopy^52^ package. The optimized structures obtained with each of the models were placed in a large cubic box, and each of the atoms was displaced along the three Cartesian directions by a small distance of ±10^–2^ Å. Forces from single point calculations on the displaced structures were then used to derive the harmonic force constants. These were then used to construct the dynamical matrix (mass-weighted Hessian), which was finally diagonalized to obtain the normal modes and associated frequencies. The finite-difference method implemented in Phonopy was used here because analytical second derivatives are currently not available in the DL_FFLUX code. Table 2 compares the calculated frequencies for the dimer using the direct- and transfer-learned dimer models to the B3LYP/6-31+G(d,p) frequencies. Assignments of the vibrational modes are provided in Table S4.1 of Section 4 of the Supporting Information, and animations showing the atomic motion (GIF) are provided as part of the data set associated with this work. The calculated frequencies for the monomer obtained from the equivalent monomer models are compared to the training level of theory in Table S3.1 of Section 3 of the Supporting Information.

**Table 2:** Calculated Vibrational Frequencies (cm^–1^) of the Formamide Dimer from the Direct- and Transfer-Learned Dimer Models^a^

mode	B3LYP	FFLUX direct	Δ	FFLUX transfer	Δ
1	63.61	59.79	3.82	55.00	8.61
2	137.39	133.50	3.89	139.00	1.61
3	145.83	142.56	3.27	143.78	2.05
4	171.55	165.56	5.99	170.25	1.30
5	178.90	180.79	1.89	179.01	0.11
6	215.04	210.49	4.55	213.27	1.77
7	492.73	459.88	32.85	444.93	47.80
8	503.43	497.96	5.47	480.46	22.97
9	609.42	610.05	0.63	608.61	0.81
10	631.35	623.58	7.77	628.36	2.99
11	825.54	769.94	55.60	766.37	59.17
12	864.14	821.00	43.14	820.91	43.23
13	1049.23	1029.44	19.79	1021.60	27.63
14	1058.91	1043.27	15.64	1051.36	7.55
15	1096.75	1096.17	0.58	1086.08	10.67
16	1103.66	1097.52	6.14	1092.40	11.26
17	1334.23	1291.09	43.14	1242.18	92.05
18	1347.53	1321.20	26.33	1276.70	70.83
19	1422.00	1399.48	22.52	1394.17	27.83
20	1422.24	1423.31	1.07	1404.44	17.80
21	1644.50	1621.77	22.73	1595.27	49.23
22	1651.65	1642.61	9.04	1629.97	21.68
23	1750.48	1770.02	19.54	1732.29	18.19
24	1780.53	1787.54	7.01	1767.84	12.69
25	2999.16	2971.82	27.34	2906.11	93.05
26	3002.21	2979.35	22.86	2984.54	17.67
27	3293.57	3258.34	35.23	3242.56	51.01
28	3338.99	3396.20	57.21	3360.47	21.48
29	3683.01	3617.59	65.42	3560.78	122.23
30	3683.49	3634.32	49.17	3624.55	58.94

a Absolute differences (Δ) from the vibrational frequencies calculated using the B3LYP/6-31+G(d,p) training level of theory are also given.

The two models are reasonably capable of recovering the frequencies predicted by the training level of theory, with mean absolute errors of 20.7 and 30.8 cm^–1^, respectively, for the direct- and transfer-learned models. The maximum errors in the calculated vibrational frequencies correspond to an energy error of less than 1.5 kJ mol^–1^, which is again lower than the commonly used chemical accuracy threshold. While this error is larger than in the models from Kamath *et al.*, where errors of the order of 1 cm^–1^ were shown to be possible,^53^ those models are designed specifically to reproduce the vibrational frequencies. The transfer-learned model is generally consistent with its direct-learned counterpart, with the largest difference between the two models being 65.71 cm^–1^ for the CH stretch vibration for which the training level of theory predicts a frequency of 2999.16 cm^–1^ (see Table 2).

Taking the Fourier transform of the autocorrelation function of the total system’s dipole moment during an MD simulation gives the IR spectrum and additionally predicts the intensities of the IR active modes according to 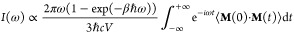
14

Here, ω is a frequency, β = 1/*k*_B_*T*, where *k*_B_ is the Boltzmann constant and *T* is the absolute temperature, *ℏ* is the reduced Planck constant, *c* is the speed of light in a vacuum, and *V* is the volume of the simulation cell. **M**(*t*) is a vector calculated from the sum of the atomic dipole moments, plus the charge transfer dipole moments calculated as the product of the atomic charge and position of every atom in the simulation box.

A quantum correction factor, *Q*_cf_, is often applied to obtain a better representation of the experimental frequencies, although the choice of this correction is arbitrary.^54^ Here, the following correction is used
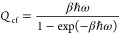
15

This functional form was chosen for ease of implementation, as it effectively means that the autocorrelation function of the total system dipole moment is simply multiplied by ω^2^.

To calculate the IR spectrum of the formamide dimer, we ran a series of 50 MD simulations at 50 K, each of 100 ps length and starting from the B3LYP/6-31+G(d,p)-optimized dimer with random initial velocities drawn from a Maxwell–Boltzmann distribution. The IR spectra from all 50 trajectories were then averaged to obtain the final simulated IR spectrum. An example of one of these dimer model trajectories (free from any nonbonded potential) is provided as a video file (MP4) in the data set associated with this work (see data availability statement for details).

**Figure 5:**
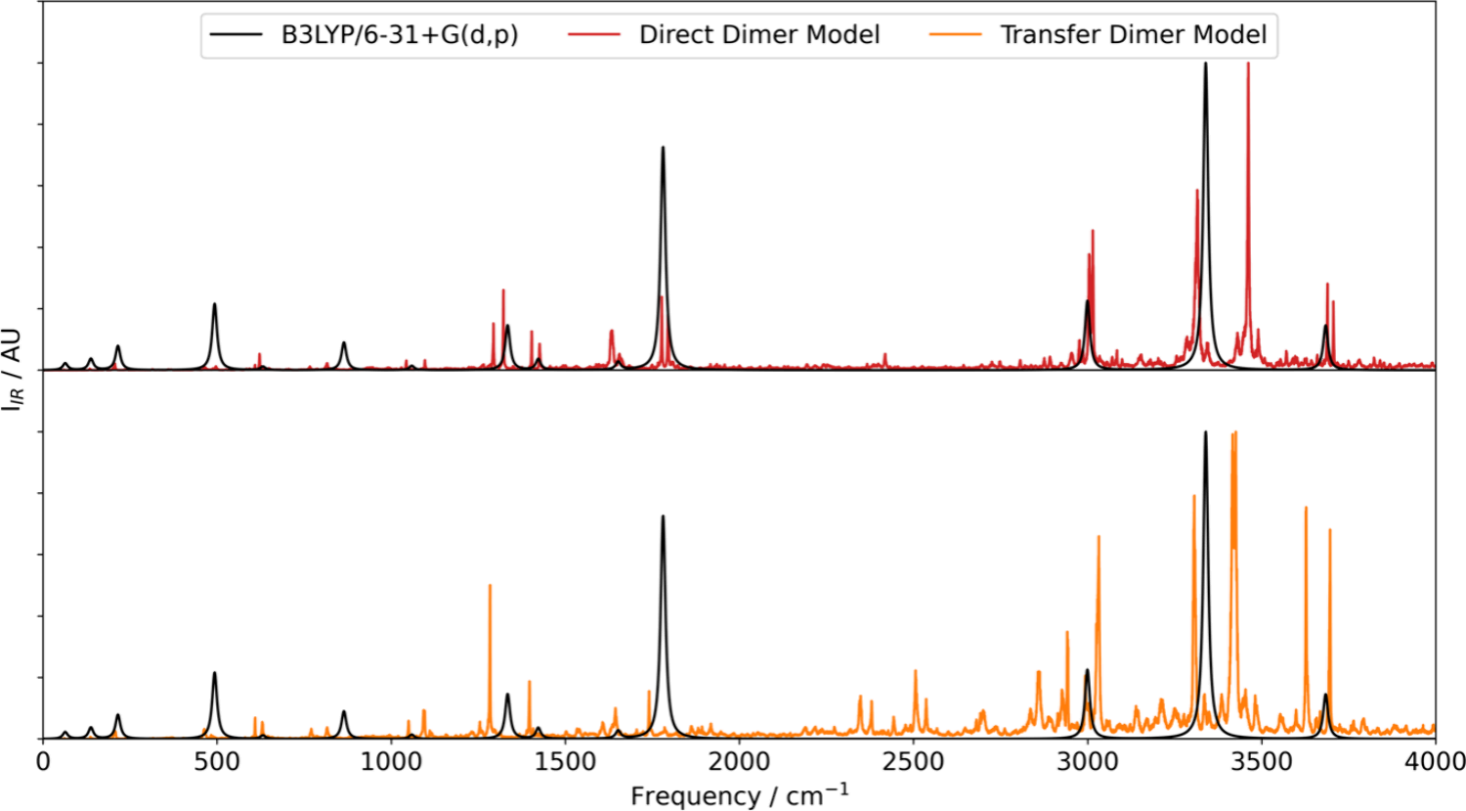
IR spectra of the formamide dimer calculated using the direct-learned dimer model (red) and the transfer-learned model (orange) and compared to the B3LYP/6-31+G(d,p) spectrum (black). A nominal Lorentzian line width of 16.7 cm^–1^ was used to generate the B3LYP spectrum.

To calculate the IR spectrum using eq 14, the atomic charges and atomic dipole moments are required to obtain the total system dipole moment. Thus, simulations were run at *L*′ = 1, although the dipole moments are not otherwise used in the simulations because all the intermolecular electrostatic interactions are captured by the atomic energy models themselves. The quantity *L*′ refers to the maximum multipolar rank present in a simulation such that *L*′ = 1 corresponds to interactions between charges (*l* = 0) between dipole moments (*l* = 1) and also between charges and dipole moments. Figure 5 compares the simulated IR spectra of the dimer obtained from the direct- and transfer-learned models with the B3LYP/6-31+G(d,p) IR spectrum.

The averaged spectra obtained from the two models show significant differences, with the transfer-learned model producing a visibly noisier spectrum. This observation can potentially be attributed to the larger errors in the total system dipole moment in the transfer-learned model. This finding is consistent with the direct-learned model predicting relative intensities closer to the training level of theory. Further support comes from the fact that the monomer models, for which the errors in the direct- and transfer-learned models are more similar, predict similar spectra (Figure S3.28 in Section 3 of the Supporting Information).

The most significant difference between the two models is the (relative) intensity of the peak at approximately 3460 cm^–1^. Peaks at frequencies above ∼3000 cm^–1^ correspond to in-phase and out-of-phase symmetric and asymmetric N–H stretches in the dimer, and the different relative phases of the atomic motion in the vibrations lead to different changes in the total system dipole moment. Errors in the models may mean that differences in the changes to the total system dipole moment are not captured perfectly, leading to the overprediction of the intensities. The MD approach to predicting IR spectra can account for anharmonic motions in the molecule that can affect the calculated vibrational frequencies. These motions are not captured in either the finite-difference approach or the calculation of the reference B3LYP/6-31+G(d,p) frequencies where the harmonic approximation is used. Therefore, differences are possible between these two methods and the MD due to the anharmonicity that the MD is able to capture. However, in practice, running the simulations at low temperature (50 K) results in minimal differences in the band positions.

Overall, despite the differences to the training level of theory, the direct- and transfer-learned models produce spectra that are reasonably consistent with each other. In future, considering the accuracy with which transfer-learned models predict molecular/system dipole moments, as well as system charges and energies as in the present work, may lead to more informed choices of the η and ζ parameters that allow the transfer-learned models to predict less noisy IR spectra.

### 4.2 Dimer Model versus Monomer Model

#### 4.2.1 Lennard-Jones Parameters

In FFLUX, a monomeric model means that a formamide molecule only “knows” about itself and can only interact electrostatically with other molecules. Monomeric models have no mechanism to predict intermolecular repulsion (nor dispersion). Only when another “body” (i.e. molecule) shares an oligomer wave function can a GPR model capture these non-electrostatic intermolecular effects. On the other hand, the dimer models can predict intermolecular repulsion, and we have shown that dispersion can potentially be accounted for by using electron correlation energies,^55^ although this requires high-level correlated wave functions that were deemed too costly for the present proof-of-concept study.

In this work, to model repulsion and dispersion with the monomeric model we use a Lennard-Jones potential of the form
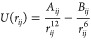
16where *r* is the separation between atoms *i* and *j*, and *A* and *B* are parameters that control the repulsive and dispersive interactions, respectively. Since the dimer models formally contain no measure of dispersion, by virtue of the chosen training level of theory, for a fair comparison between the monomer and dimer models we set the *B* parameter to zero.

The dimer model is able to predict the “exact” intermolecular electrostatic energy, as this contribution is built into the  terms that the models are trained on (see eqs 2 and 4), whereas in the monomeric model, the intermolecular electrostatics are determined from the predicted atomic multipole moments of the two monomers. The hexadecapole moments are the highest rank multipole moments that can be predicted in DL_FFLUX calculations, and assuming well-converged electrostatics, this high rank should ensure that the monomer and dimer model calculations are as consistent as possible. The intermolecular multipolar electrostatics in DL_FFLUX are controlled by the parameter *L*′, which represents the maximum multipolar rank present in a simulation, as noted above. A value of *L*′ = 4 means that the atomic charges, dipole, quadrupole, octupole, and hexadecapole moments, and all the interactions between them are included. While the transferability of the monomer moments to the dimer system is questionable, due to cancellation of errors, *L*′ = 4 provides a reasonable representation of the “true” intermolecular atom–atom electrostatic energies in the dimer (assessed in Figure S5.1 of Section 5 of the Supporting Information).

The Lennard-Jones parameters used in our previous FFLUX calculations on formamide^25^ were adapted for *L*′ = 4 calculations by running a series of geometry optimizations on the formamide dimer with the previous parameters scaled by a factor *n* to obtain scaled parameters, , as
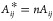
17where *n* was varied from 70 to 130% in steps of 2.5%, and geometry optimizations of the dimer were carried out as described in Section 3.2 with each parameter set. We then calculated the RMSDs of the final geometries relative to the B3LYP/6-31+G(d,p)-optimized dimer and selected the parameter set with the smallest RMSD for use in dimer simulations using the monomeric model. The optimized Lennard-Jones parameters and RMSDs for each parameter set tested are given in Section 6 of the Supporting Information.

#### 4.2.2 Geometry Optimizations

The optimization process described in Section 4.2.1 was repeated with the direct-learned formamide monomer model with *L*′ = 4 and the optimized Lennard-Jones parameters, and the results were compared to those obtained with the direct-learned dimer model. To compare the accuracy of the energetics obtained using the monomeric and dimeric models, we compare the predicted formation energies *E*_form_:
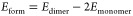
18where *E*_dimer_ is the average dimer energy calculated using either the dimer model, or the monomer model with Lennard-Jones parameters, across the 30 dimer geometry optimizations, and *E*_monomer_ is the average energy of the FFLUX-optimized monomer, from the monomer model, across the 12 monomer optimizations. This comparison is reasonable because both the monomer and dimer models are trained from data at the same B3LYP/6-31+G(d,p) level of theory, and their energies are therefore compatible. Errors on the calculated *E*_form_ were estimated from the standard deviations of the energies from the two sets of geometry optimizations but were found to be the order of 10^–4^ kJ mol^–1^ and were therefore considered negligible. Table 3 shows the formation energies and RMSD of the optimized geometries obtained using the dimer and monomer models for the dimer.

**Table 3:** Comparison of the Formamide Dimer Formation Energy Calculated at the B3LYP/6-31+G(d,p) Training Level of Theory and Using the Dimer Energies from the FFLUX Monomer and Dimer GPR Models Together with Monomer Energies from the Monomer Models^a^

**model**	**formation energy/kJ mol**^**–1**^	**RMSD**_**Avg**_**/Å**	**σ**_**RMSD**_**/Å**
B3LYP/6-31+G(d,p)	–56.9		
monomer direct + LJ	–43.4	0.05	1.1 × 10^–4^
dimer direct	–56.1	0.01	2.3 × 10^–4^

a The Lennard-Jones potential used for calculation of the dimer energy with the monomer model only included a repulsive contribution for fairer comparison to B3LYP, which has no measure of dispersion. The RMSD and standard deviation in the FFLUX-optimized dimers to the optimized structure from the training level of theory are also shown for comparison.

The calculated formation energies show that the dimer model offers a significant improvement over the monomer model with the Lennard-Jones parametrization of repulsion only, with sub-kJ mol^–1^ accuracy in contrast to an error of 13.5 kJ mol^–1^. Although the formation energy calculated with the monomer model could be improved by adjusting the parameters in the Lennard-Jones potential, doing so may mean a correct result is obtained for the wrong reasons. On the other hand, when combining the monomer and dimer GPR models, all the energetic information is derived minimally and indeed directly from quantum mechanics. Parametrization then becomes unnecessary, an advantage that is the driver for the current work.

**Figure 6:**
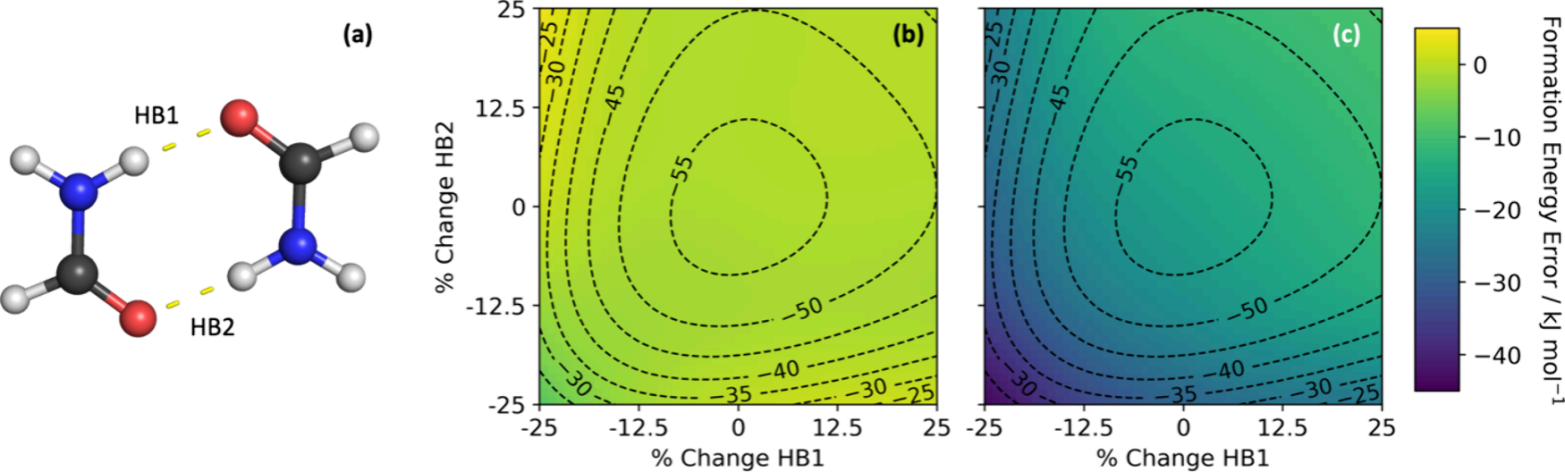
Calculated formation energies *E*_form_ of formamide dimers with the hydrogen bond lengths HB1 and HB2, shown in panel (a), distorted by ±25%, with the dimer energies calculated using (b) the dimer model and (c) the monomer model with only repulsive Lennard-Jones parameters (i.e., the *B* parameter in the potential was set to 0). On each of the plots, the B3LYP/6-31+G(d,p) values are shown by contours lines from −25 to −55 kJ mol^–1^ in steps of 5 kJ mol^–1^. The heatmaps beneath the contours show the errors in the formation energies relative to the reference *E*_form_ obtained at the training level of theory.

To further test the models, a series of distorted dimers were produced by compressing and expanding the two hydrogen bonds in the formamide dimer by 25%, in steps of 1%, to produce 2601 distorted geometries (51 displacements of bond 1 × 51 displacements of bond 2). The energies for forming these distorted dimers were calculated using both the dimer and monomer models for the dimer and compared to *E*_form_ calculated at the training level of theory. The error for the two models is shown in Figure 6 as a heatmap, with the B3LYP values overlaid as contours.

The errors once again show that the dimer model offers significantly higher accuracy than the monomer model combined with Lennard-Jones parameters, with the dimer model having a maximum absolute error of 7.5 kJ mol^–1^ compared to 43.2 kJ mol^–1^ for the monomer model with parametrized repulsion. For the dimer model, the maximum errors correspond to geometries with compressed hydrogen bonds, which can be explained by the fact that such geometries are unlikely to be adequately covered by the training set.

#### 4.2.3 Vibrational Frequencies and IR Spectra

We also compared the calculated vibrational frequencies of the formamide dimer obtained using the direct-learned dimer model and the direct-learned monomer model with parametrized repulsion to the training level of theory (Table 4).

**Table 4:** Vibrational Frequencies (cm^–1^) of the Formamide Dimer Calculated Using the Direct-Learned Monomer Model with Parametrized Repulsion and the Direct-Learned Dimer Model^a^

mode	B3LYP	monomer model	Δ	dimer model	Δ
1	63.61	73.44	9.83	59.79	3.82
2	137.39	143.81	6.42	133.50	3.89
3	145.83	157.69	11.86	142.56	3.27
4	171.55	163.66	7.89	165.56	5.99
5	178.90	190.69	11.79	180.79	1.89
6	215.04	206.26	8.78	210.49	4.55
7	492.73	454.55	38.18	459.88	32.85
8	503.43	478.35	25.08	497.96	5.47
9	609.42	629.18	19.76	610.05	0.63
10	631.35	661.63	30.28	623.58	7.77
11	825.54	893.44	67.90	769.94	55.60
12	864.14	929.22	65.08	821.00	43.14
13	1049.23	1038.65	10.58	1029.44	19.79
14	1058.91	1054.74	4.17	1043.27	15.64
15	1096.75	1093.57	3.18	1096.17	0.58
16	1103.66	1104.18	0.52	1097.52	6.14
17	1334.23	1334.62	0.39	1291.09	43.14
18	1347.53	1344.09	3.44	1321.20	26.33
19	1422.00	1425.00	3.00	1399.48	22.52
20	1422.24	1433.85	11.61	1423.31	1.07
21	1644.50	1692.69	48.19	1621.77	22.73
22	1651.65	1700.01	48.36	1642.61	9.04
23	1750.48	1775.76	25.28	1770.02	19.54
24	1780.53	1794.51	13.98	1787.54	7.01
25	2999.16	3072.66	73.50	2971.82	27.34
26	3002.21	3075.22	73.01	2979.35	22.86
27	3293.57	3403.08	109.51	3258.34	35.23
28	3338.99	3414.87	75.88	3396.20	57.21
29	3683.01	3691.52	8.51	3617.59	65.42
30	3683.49	3691.60	8.11	3634.32	49.17

a Absolute differences (Δ) from the vibrational frequencies calculated at the B3LYP/6-31+G(d,p) training level of theory are given for comparison.

The vibrational frequencies predicted by the monomer model are generally less accurate, with a mean absolute error of 27.47 cm^–1^ compared to 20.65 cm^–1^ for the dimer model. This difference is approximately equivalent to 0.08 kJ mol^–1^. While this is quite small, the dimer model also has the benefit of practicality by virtue of not requiring parametrization of a nonbonded potential. This emphasizes the potential plug-and-play nature of FFLUX.

As expected, we find that the in-phase and out-of-phase C=O···HN hydrogen bond stretches at 171.55 and 215.04 cm^–1^, respectively (modes 4 and 6), are predicted more accurately with the dimer model. These intermolecular stretches are influenced by both the intermolecular electrostatics and the parametrized repulsion. Hence, we tentatively attribute the poorer performance of the monomer model to the Lennard-Jones parameters, which were optimized to obtain the geometry of the dimer, not transferring well to predicting vibrational frequencies. The first six modes are predominantly intermolecular in nature and are therefore better predicted by the dimer model, which has been trained on these intermolecular interactions.

Several of the (predominantly) intramolecular modes involving H-bonded atoms are also better described by the dimer model. These include the symmetric and asymmetric C=O stretches (modes 23 and 24) and the in-phase and out-of-phase symmetric NH stretches (modes 27 and 28). The ability of the dimer model to better reproduce the majority of the intramolecular modes is particularly noteworthy. It is known that upon formation of hydrogen bonds, some vibrations are red-shifted^56^ and blue-shifted^57^ due to intermolecular polarization, and the monomer model cannot capture this effect, while the dimer model can. For example, the symmetric NH stretch is expected to be red-shifted upon formation of hydrogen bonds. At the training level of theory, the stretch occurs at ∼3590 cm^–1^ in the monomer and is red-shifted to ∼3300 cm^–1^ in the dimer (modes 27 and 28). The monomer model predicts these modes with large errors (109.51 and 75.88 cm^–1^), but the dimer model has smaller errors of 35.23 and 57.21 cm^–1^, respectively, showing its ability to better account for intermolecular polarization. However, the dimer model does not appear to capture the shift in the asymmetric stretch as effectively, with a significantly larger error than the monomer model. This could be a consequence of the higher dimensionality of the dimer model. There are 3*N* – 6 ALF features in the GPR models used in FFLUX simulations, where *N* is the number of atoms. Larger systems have higher dimensional feature spaces and are therefore more difficult to model, meaning that they can be more prone to error. Despite this, the monomer model performs better for only 7 of the 30 modes, indicating that the dimer model generally provides an improved description of the vibrational modes.

**Figure 7:**
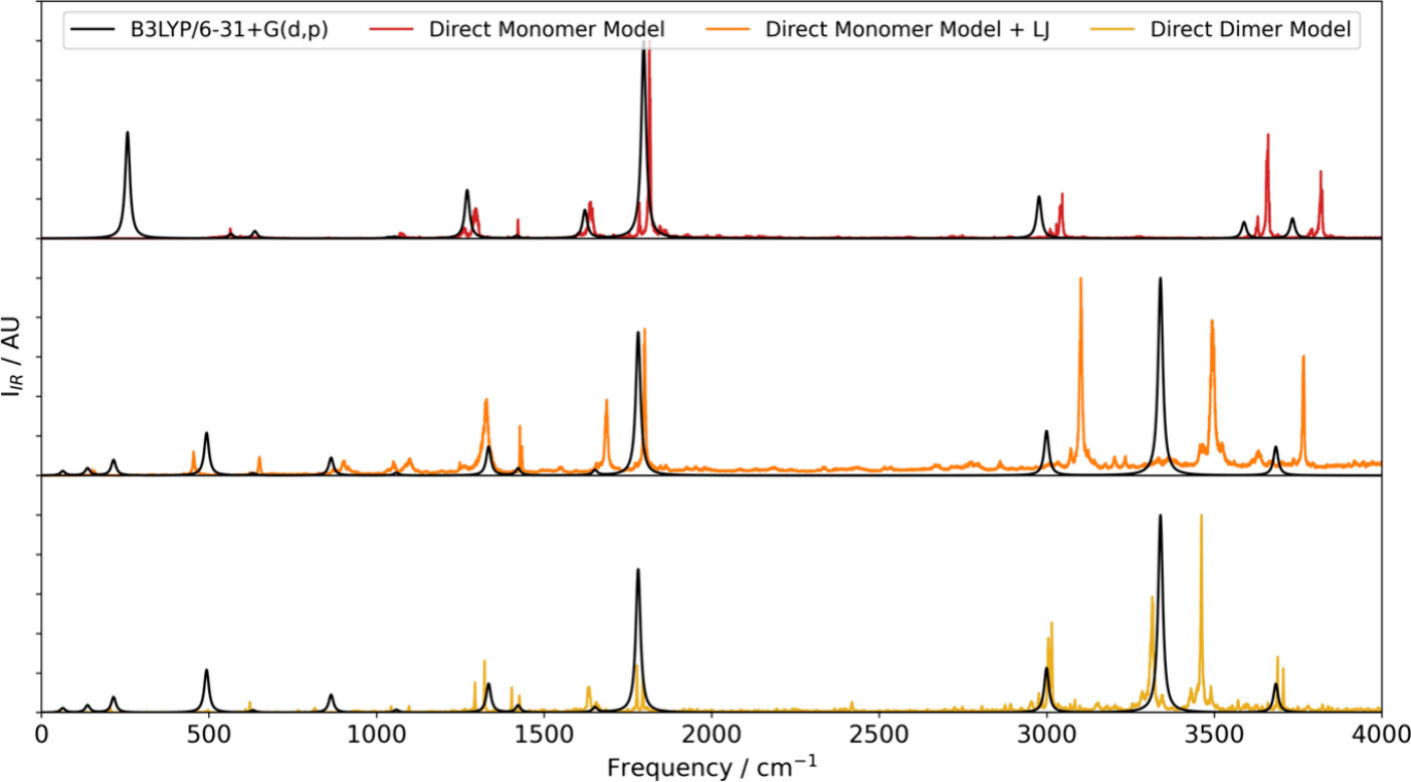
IR spectra of the formamide dimer calculated using the direct-learned monomer model with parametrized repulsion (orange, middle) and the direct-learned dimer model (yellow, bottom) compared to the B3LYP/6-31+G(d,p) spectrum (black). To aid the discussion, the spectrum of the formamide monomer (red, top) obtained with the monomer model is also compared to the corresponding B3LYP/6-31+G(d,p) spectrum (also black). A nominal Lorentzian line width of 16.7 cm^–1^ was used to generate the B3LYP spectra.

IR spectra were modeled as described in Section 4.1.3. However, for the calculations using the monomer model, a higher electrostatic rank of *L*′ = 4 was used to ensure that the electrostatic interactions reflect those in the dimer model as accurately as possible. Figure 7 compares the IR spectra obtained from the monomer and dimer models to those from the B3LYP/6-31+G(d,p) training level of theory.

The IR spectrum of the monomer is generally reproduced well by the monomeric model, yielding a good reproduction of the frequencies and relative intensities of the peaks between 1000 and 3000 cm^–1^. Outside this range, there are two notable deviations. The first is that the feature associated with NH_2_ wagging at approximately 250 cm^–1^ is not present in any of the spectra from the individual trajectories used to calculate the average. The second is that the frequencies of the NH stretches between 3500 and 4000 cm^–1^ are slightly overpredicted compared to the training level of theory and the frequencies calculated using the finite-difference method.

The absence of the NH_2_ wagging peak could be attributed either to deficiencies in the sampling in the MD simulations or to errors in the calculated intensity due to issues with the GPR model itself. To investigate further, we calculated the spectral density from the Fourier transform of the velocity autocorrelation function (Figure S3.29 in Section 3 of the Supporting Information). This is roughly equivalent to a phonon density of states without weighting for changes in dipole moment as in the simulated IR spectra. The spectrum shows a clear peak at ∼250 cm^–1^, confirming that the motion is sampled in the MD simulations. This indicates that the issue is in the prediction of the intensity of the NH_2_ wagging mode and thus that the GPR model does not adequately capture the change in polarization associated with this mode.

As the MD simulations can potentially capture anharmonic effects, comparison was also made to the anharmonic spectra calculated using GAUSSIAN (Figures S7.1 and S7.2 in Section 7 of the Supporting Information). We found better agreement between the FFLUX spectra and the harmonic B3LYP spectra, indicating that anharmonic effects are not prominent at the low temperatures at which the MD simulations were performed. With this in mind, we note that the monomer simulations, and the dimer simulations with the dimer model, depend only on the GPR model, so errors in the frequencies must be attributable to the model and not to any issues in the parametrization of intermolecular interactions.

Comparing the dimer and monomer IR spectra suggests that the peaks associated with the NH stretches should be red-shifted in the dimer due to the hydrogen bonding. It is pleasing that this effect is clearly seen in the reference B3LYP/6-31+G(d,p) spectra. The monomer model with Lennard-Jones repulsion partially captures this effect due to the ability of the geometry-dependent multipole moments to capture some level of intramolecular polarization. However, the peaks associated with the NH vibrations are predicted to occur at higher frequencies than by the training level of theory. Using the dimer model to calculate the spectrum predicts the red-shift with greater accuracy, which strongly suggests that some of the intermolecular polarization captured naturally by the dimer model is missed by the monomeric model. While force fields can capture intermolecular polarization using polarizability parameters, choosing and optimizing these, as for the Lennard-Jones parameters used to describe repulsion in the monomer model, can be challenging. The use of oligomeric models, such as the dimer model in FFLUX, removes the ambiguity associated with this process by describing intermolecular polarization in a manner that is consistent with quantum mechanics.

## 5 Conclusions

One of the main aims of the FFLUX force field is to be able to perform simulations with quantum mechanical levels of accuracy at a comparable cost to a traditional force field. In this work, we have addressed accuracy by integrating intermolecular repulsion into the Gaussian process regression (GPR) models. We showed that it is possible to use, for the first time, dimeric GPR models in FFLUX simulations, and that doing so yields improved accuracy compared to monomeric models with intermolecular multipolar electrostatics and parametrized repulsion. Although we did not explicitly account for dispersion in this proof-of-concept study, we have previously shown that dynamic electron correlation can also be machine-learned.^55^ These dispersion-aware GPR models can be easily incorporated into the FFLUX workflow but have yet to be used in simulations.

This work has demonstrated three key advantages of oligomeric models over the monomeric models with Lennard-Jones potentials used in previous FFLUX simulations. The first is practicality for the user, as the time-consuming (and possibly error-prone) parametrization of nonbonded potentials is no longer required. The second is the avoidance of ambiguity from the generally high sensitivity of simulations to the nonbonded parameters and the likely possibility that potentials can be tailored to obtain the “right result” for the wrong physical reasons. The third and final advantage is that the FFLUX simulations lie closer to the quantum mechanical reality.

The latter advantage was particularly evident in this work in the context of geometry optimizations, where the dimer model was able to more accurately capture the geometry predicted by the training level of theory, and simulated vibrational frequencies and infrared spectra, where the improved description of intermolecular polarization was visible through the red-shifting of the peaks associated with NH vibrations and more accurate relative band intensities.

A possible downside to using oligomeric models is the increased time required for training due to the higher dimensionality. In this work, we have demonstrated that this can be mitigated using transfer learning. With appropriate parameters, the training of both the monomer and dimer models can both be sped up by approximately an order of magnitude while maintaining similar errors to direct-learned models. This work, in part, acts as an introduction to the use of transfer-learned models in FFLUX simulations, and this approach will be explored in greater detail in imminent work. Even in cases where monomeric and oligomeric models produce similar results, the ease of use of an oligomeric model, in particular by avoiding the need to parametrize a nonbonded potential, may still outweigh the increased training demand.

Finally, a dimer was chosen as the simplest oligomer for this proof-of-concept study, but the two-body effects learned in the models may not be suitable for application to larger systems like molecular crystals where *N*-body effects (*N* > 2) may be important. We will address this matter in more detail in future work. However, at present, DL_FFLUX only allows oligomeric models to be used for simulations of the oligomers they are trained for, and the significant changes required for simulations on larger systems represent a longer-term goal.

## Data Availability

Multimedia: animations of the formamide dimer vibrational modes obtained with B3LYP/6-31+G(d,p) (GIF) and a video of one of the MD trajectories run with the formamide dimer model at 50 K to generate a simulated IR spectrum (MP4). The data supporting the findings in this paper are available free of charge from the “Data for: Incorporating Non-Covalent Interactions in Transfer Learning Gaussian Process Regression Models for Molecular Simulations” repository at https://research.manchester.ac.uk/en/datasets/data-for-incorporating-non-covalent-interactions-in-transfer-lear.

## Supporting Information Available

Section 1: distribution of wave function energies in the monomer and dimer data sets (Figure S1.1) and distributions of atomic energies and charges in the monomer and dimer data sets (Figures S1.2–S1.5); Section 2: comparison of transfer-learned models trained using source models with training data selected using random and passive sampling (Tables S2.1 and S2.2); Section 3: predicted molecular properties and training times for transfer-learned monomer models (Figure S3.1), S-curves showing the prediction errors in the IQA energies (Figure S3.2), and multipole moments (Figures S3.3–S3.27) from the direct- and transfer-learned monomer models, comparison of vibrational frequencies calculated for the monomer using the direct- and transfer-learned models (Table S3.1), and simulated infrared spectra for the monomer calculated using the direct- and transfer-learned models compared to spectra obtained with the B3LYP/6-31+G(d,p) training level of theory (Figure S3.28); Section 4: assignment of the vibrational modes of the formamide dimer (Table S4.1); Section 5: heatmaps showing the errors in the intermolecular atom–atom electrostatic energies calculated for a formamide dimer and using the multipole moments from formamide monomers (Figure S5.1); Section 6: initial nonbonded parameters used in the scaling tests (Table S6.1) and root-mean-square deviation in the structure of the optimized formamide dimer relative to the B3LYP/6-31+G(d,p) training level of theory obtained with each scaled parameter set (Table S6.2); Section 7: comparison of the FFLUX monomer and dimer IR spectra to harmonic and anharmonic spectra calculated at the training level of theory (Figures S7.1 and S7.2) (PDF)
