# Supplementary material for: Incorporating Noncovalent Interactions in Transfer Learning Gaussian Process Regression Models for Molecular Simulations
Source: J Chem Theory Comput. 2024 Jul 9;20(14):5994–6008. doi: 10.1021/acs.jctc.4c00402 (PMC11270819; doi:10.1021/acs.jctc.4c00402)
Supplement: Supplementary file 1 — ct4c00402_si_001.pdf [file ct4c00402_si_001.pdf]

Supporting Information

Incorporating Non-Covalent Interactions in Transfer Learning Gaussian Process Regression Models for Molecular Simulations

Matthew L. Brown, Bienfait K. Isamura, Jonathan M. Skelton and Paul L. A. Popelier*

Department of Chemistry, The University of Manchester, Oxford Road, Manchester, M13 9PL, United Kingdom

*Phone: +44 161 3064511. Email: pla@manchester.ac.uk

Contents

1 Energy Distributions in Hybrid Datasets S2

2 Transfer Learnt Models Using Passive Sampling S4

3 Evaluation of Monomer Model S7

4 Assignment of the Formamide Dimer Vibrational Modes S21

5 Transferability of Monomer Multipole Moments S22

6 Optimisation of Lennard-Jones Parameters for Monomer Model S24

7 Anharmonic Infrared Spectra S26

References S27

1 Energy Distributions in Hybrid Datasets

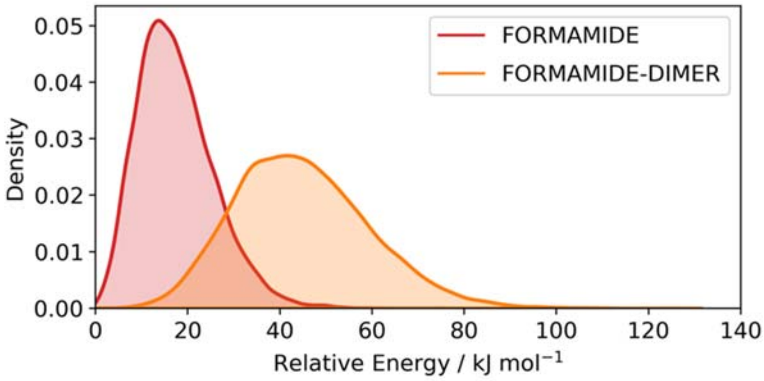

Figure S1.1. Distribution of wavefunction energies present in the datasets for the formamide monomer (red) and dimer (orange).

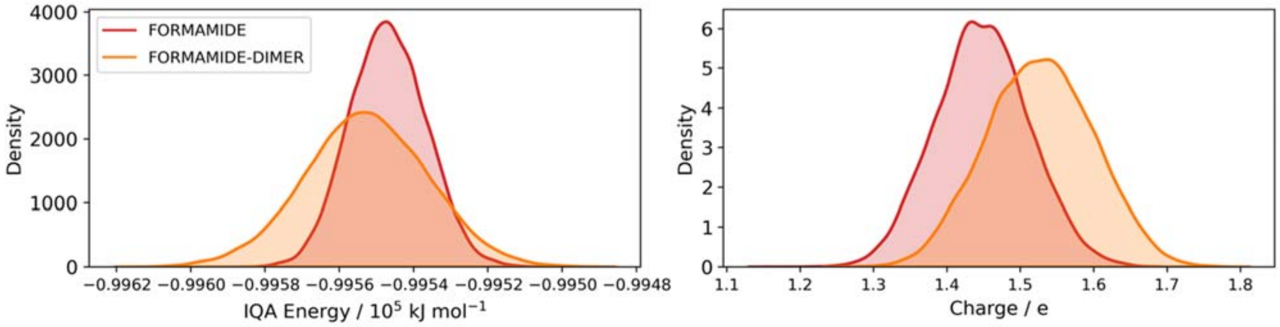

Figure S1.2. Distribution of IQA energies and charges on the C atom(s) in the datasets for the formamide monomer (red) and dimer (orange).

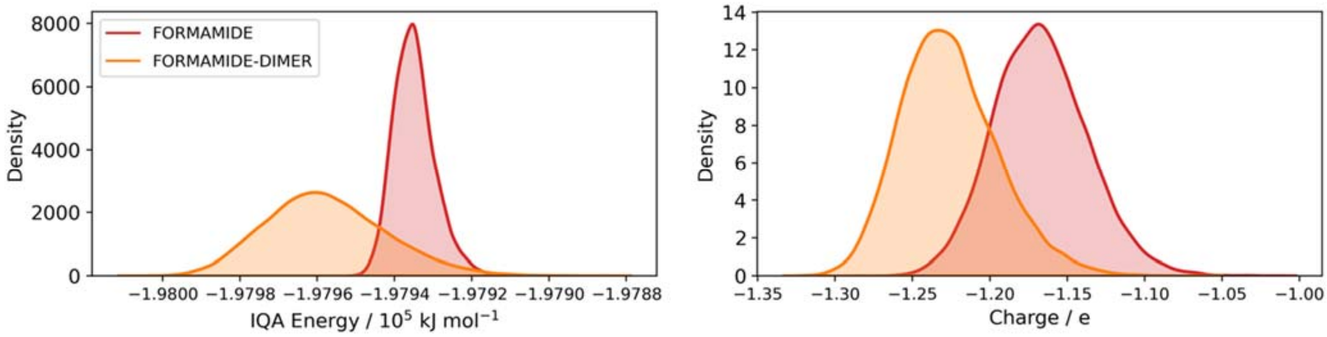

Figure S1.3. Distribution of IQA energies and charges on the O atom(s) in the datasets for the formamide monomer (red) and dimer (orange).

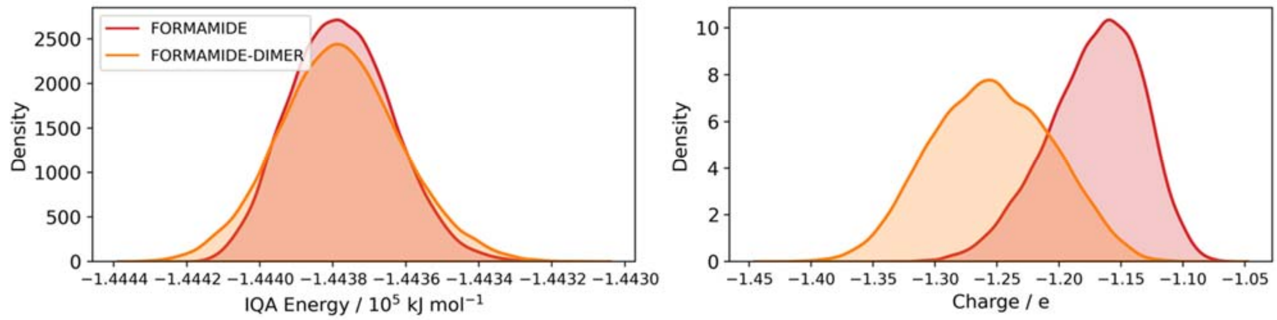

Figure S1.4. Distribution of the IQA energies and charges on the N atom(s) in the datasets for the formamide monomer (red) and dimer (orange).

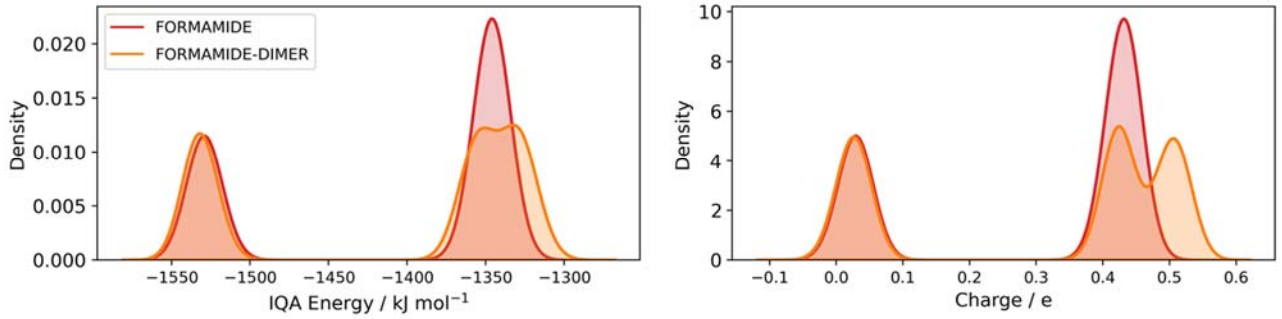

Figure S1.5. Distribution of IQA energies and charges on the H atom(s) in the datasets for the formamide monomer (red) and dimer (orange).

2 Transfer Learnt Models Using Passive Sampling

In the transfer learning implemented in our in-house machine learning engine, FEREBUS,[1-3] a "source" model is constructed from the dataset, with the number of points controlled by the knowledge compression coefficient, η (eq 10 in the main text). These "source" points are a sample of the training set of the "target" model which, in this case, contains all 5,000 training points. For the models presented in the main text and Section 3 of the Supporting Information (SI) these points are chosen using random sampling. Using a random sample means models may not be generated consistently, and multiple models are possible for a given η and relaxation weight ζ.

One way to generate more consistent models is to use an enhanced sampling technique when constructing the source model. In this section, we report complementary results obtained using passive sampling,[4] which aims to select the most diverse points from the target dataset. A series of transfer learnt formamide monomer models were generated using both random and passive sampling to construct the source model. These models used $\eta = 0.01$ and $\eta = 0.1$, both with $\zeta = 0.01$. For each of the two sets of η and ζ, 8 transfer learnt models were generated, 4 using random sampling for generating the source model, and 4 using passive sampling. The root-mean-square-error (RMSE), mean absolute error (MAE), maximum absolute error (maxAE) and mean absolute percentage error (MAPE) for each atom in the formamide monomer are given in Table S2.1 ($\eta = 0.01$) and Table S2.2 ($\eta = 0.1$).

For the models with a lower η (0.01), the source models constructed with passive sampling tend to lead to target models that are more consistent than the random models because the error metrics for the majority of the atoms show smaller standard deviations. For the models trained with the larger source models ($\eta = 0.1$), the standard deviations in the model RMSEs and MAEs tend to be more similar between source models constructed with random and passive sampling models, with only the maxAE being improved by the use of passive sampling (except for H6). A more detailed assessment of the impact of the source model sampling on the target model will be performed in future work.

Table S2.1. Transfer-learnt formamide monomer models generated using $\boldsymbol{\eta} = \mathbf{0.01}$ and $\zeta = 0.01$ with source models generated using random and passive sampling. The RMSE, MAE and maxAE are expressed in kJ mol^{-1} while the MAPE is given in %. The mean and standard deviation σ of each metric across multiple training runs is also given.

		Random						Passive					
Atom	Parameter	Run 1	Run 2	Run 3	Run 4	Mean	σ	Run 1	Run 2	Run 3	Run 4	Mean	σ
C1	RMSE	0.562	0.229	0.237	0.245	0.318	0.163	0.248	0.249	0.235	0.242	0.244	0.007
	MAE	0.451	0.182	0.187	0.194	0.254	0.132	0.195	0.195	0.186	0.193	0.192	0.005
	maxAE	4.031	0.792	0.928	1.023	1.694	1.561	1.680	1.274	0.895	0.911	1.190	0.371
	MAPE	4.5×10^{-4}	1.8×10^{-4}	1.9×10^{-4}	1.9×10^{-4}	2.5×10^{-4}	1.3×10^{-4}	2.0×10^{-4}	2.0×10^{-4}	1.9×10^{-4}	1.9×10^{-4}	1.9×10^{-4}	4.6×10^{-6}
O2	RMSE	0.014	0.012	0.016	0.022	0.016	0.004	0.033	0.044	0.027	0.014	0.029	0.012
	MAE	0.010	0.009	0.011	0.016	0.011	0.003	0.024	0.032	0.019	0.010	0.021	0.009
	maxAE	0.078	0.056	0.105	0.104	0.086	0.024	0.334	0.520	0.208	0.120	0.296	0.173
	MAPE	5.1×10^{-6}	4.5×10^{-6}	5.6×10^{-6}	7.9×10^{-6}	5.8×10^{-6}	1.5×10^{-6}	1.2×10^{-5}	1.6×10^{-5}	9.8×10^{-6}	5.1×10^{-6}	1.1×10^{-5}	4.6×10^{-6}
N3	RMSE	0.110	0.092	0.088	0.092	0.095	0.010	0.099	0.222	0.092	0.092	0.126	0.064
	MAE	0.087	0.072	0.067	0.072	0.074	0.009	0.078	0.181	0.070	0.072	0.101	0.054
	maxAE	0.456	0.346	0.451	0.397	0.413	0.052	0.393	1.144	0.402	0.378	0.579	0.377
	MAPE	6.0×10^{-5}	5.0×10^{-5}	4.6×10^{-5}	5.0×10^{-5}	5.2×10^{-5}	6.0×10^{-6}	5.4×10^{-5}	1.3×10^{-4}	4.9×10^{-5}	5.0×10^{-5}	7.0×10^{-5}	3.7×10^{-5}
H4	RMSE	0.006	0.006	0.076	0.006	0.024	0.035	0.067	0.050	0.006	0.038	0.040	0.026
	MAE	0.004	0.004	0.058	0.004	0.018	0.027	0.051	0.035	0.004	0.027	0.029	0.019
	maxAE	0.044	0.078	0.484	0.073	0.170	0.210	0.342	0.392	0.069	0.280	0.271	0.142
	MAPE	2.9×10^{-4}	2.7×10^{-4}	3.8×10^{-3}	2.7×10^{-4}	1.1×10^{-3}	1.7×10^{-3}	3.3×10^{-3}	2.3×10^{-3}	2.8×10^{-4}	1.8×10^{-3}	1.9×10^{-3}	1.3×10^{-3}
H5	RMSE	0.008	0.008	0.007	0.052	0.019	0.022	0.008	0.008	0.007	0.026	0.012	0.009
	MAE	0.006	0.006	0.005	0.037	0.013	0.016	0.005	0.005	0.005	0.019	0.009	0.007
	maxAE	0.054	0.082	0.055	0.286	0.119	0.112	0.088	0.053	0.061	0.153	0.089	0.046
	MAPE	4.2×10^{-4}	4.1×10^{-4}	3.8×10^{-4}	2.8×10^{-3}	1.0×10^{-3}	1.2×10^{-3}	3.9×10^{-4}	4.0×10^{-4}	4.0×10^{-4}	1.4×10^{-3}	6.5×10^{-4}	5.1×10^{-4}
H6	RMSE	0.009	0.031	0.007	0.037	0.021	0.015	0.007	0.007	0.008	0.007	0.008	0.001
	MAE	0.006	0.024	0.005	0.029	0.016	0.012	0.005	0.005	0.006	0.005	0.005	0.001
	maxAE	0.057	0.184	0.041	0.277	0.140	0.112	0.046	0.033	0.046	0.034	0.040	0.007
	MAPE	4.7×10^{-4}	1.7×10^{-3}	3.8×10^{-4}	2.2×10^{-3}	1.2×10^{-3}	9.0×10^{-4}	3.9×10^{-4}	3.9×10^{-4}	4.7×10^{-4}	3.8×10^{-4}	4.0×10^{-4}	4.1×10^{-5}

Table S2.2. Transfer-learnt formamide monomer models generated using $\boldsymbol{\eta} = \mathbf{0.1}$ and $\zeta = 0.01$ with source models generated using random and passive sampling. The RMSE, MAE and maxAE are expressed in kJ mol^{-1} while the MAPE is given in %. The mean and standard deviation σ of each metric across multiple training runs is also given.

		Random						Passive					
Atom	Parameter	Run 1	Run 2	Run 3	Run 4	Mean	σ	Run 1	Run 2	Run 3	Run 4	Mean	σ
C1	RMSE	0.243	0.264	0.244	0.248	0.250	0.010	0.237	0.275	0.243	0.244	0.250	0.017
	MAE	0.191	0.214	0.193	0.195	0.198	0.011	0.191	0.223	0.193	0.192	0.200	0.016
	maxAE	1.112	0.926	1.039	1.442	1.130	0.222	0.766	1.091	1.166	1.198	1.055	0.198
	MAPE	1.9×10^{-4}	2.1×10^{-4}	1.9×10^{-4}	2.0×10^{-4}	2.0×10^{-4}	1.1×10^{-5}	1.9×10^{-4}	2.2×10^{-4}	1.9×10^{-4}	1.9×10^{-4}	2.0×10^{-4}	1.6×10^{-5}
O2	RMSE	0.014	0.019	0.018	0.013	0.016	0.003	0.015	0.019	0.014	0.015	0.016	0.002
	MAE	0.011	0.014	0.013	0.009	0.012	0.002	0.011	0.014	0.011	0.011	0.012	0.001
	maxAE	0.065	0.197	0.108	0.052	0.105	0.066	0.063	0.160	0.073	0.097	0.098	0.044
	MAPE	5.3×10^{-6}	6.9×10^{-6}	6.6×10^{-6}	4.6×10^{-6}	5.9×10^{-6}	1.1×10^{-6}	5.7×10^{-6}	7.0×10^{-6}	5.3×10^{-6}	5.6×10^{-6}	5.9×10^{-6}	7.3×10^{-7}
N3	RMSE	0.127	0.106	0.101	0.103	0.109	0.012	0.109	0.088	0.084	0.103	0.096	0.012
	MAE	0.102	0.080	0.080	0.078	0.085	0.011	0.086	0.067	0.065	0.077	0.074	0.010
	maxAE	0.534	0.627	0.371	0.763	0.574	0.165	0.457	0.464	0.399	0.545	0.466	0.060
	MAPE	7.0×10^{-5}	5.5×10^{-5}	5.5×10^{-5}	5.4×10^{-5}	5.9×10^{-5}	7.8×10^{-6}	5.9×10^{-5}	4.6×10^{-5}	4.5×10^{-5}	5.3×10^{-5}	5.1×10^{-5}	6.7×10^{-6}
H4	RMSE	0.006	0.005	0.005	0.008	0.006	0.001	0.005	0.005	0.005	0.005	0.005	0.000
	MAE	0.004	0.004	0.004	0.006	0.004	0.001	0.004	0.004	0.004	0.004	0.004	0.000
	maxAE	0.058	0.021	0.037	0.047	0.041	0.015	0.025	0.040	0.025	0.026	0.029	0.007
	MAPE	2.8×10^{-4}	2.3×10^{-4}	2.4×10^{-4}	3.6×10^{-4}	2.8×10^{-4}	6.0×10^{-5}	2.4×10^{-4}	2.4×10^{-4}	2.5×10^{-4}	2.5×10^{-4}	2.4×10^{-4}	7.6×10^{-6}
H5	RMSE	0.007	0.007	0.006	0.007	0.007	0.000	0.008	0.008	0.008	0.007	0.008	0.000
	MAE	0.005	0.005	0.005	0.005	0.005	0.000	0.006	0.006	0.006	0.005	0.006	0.000
	maxAE	0.039	0.031	0.041	0.050	0.040	0.008	0.051	0.047	0.060	0.052	0.053	0.005
	MAPE	3.6×10^{-4}	3.5×10^{-4}	3.4×10^{-4}	3.5×10^{-4}	3.5×10^{-4}	8.2×10^{-6}	4.5×10^{-4}	4.1×10^{-4}	4.2×10^{-4}	3.8×10^{-4}	4.2×10^{-4}	2.9×10^{-5}
H6	RMSE	0.007	0.007	0.007	0.008	0.007	0.000	0.007	0.006	0.007	0.007	0.007	0.000
	MAE	0.005	0.005	0.005	0.006	0.005	0.000	0.005	0.005	0.005	0.005	0.005	0.000
	maxAE	0.038	0.031	0.032	0.036	0.035	0.003	0.028	0.028	0.036	0.035	0.032	0.005
	MAPE	4.0×10^{-4}	3.8×10^{-4}	3.6×10^{-4}	4.2×10^{-4}	3.9×10^{-4}	2.6×10^{-5}	3.5×10^{-4}	3.4×10^{-4}	3.6×10^{-4}	3.6×10^{-4}	3.5×10^{-4}	8.1×10^{-6}

3 Evaluation of Monomer Model

As described in Section 3.1.1 of the main text, the dataset for the monomer model was generated by performing a 1 ns simulation (with 1 fs timestep) at 300 K using the GAFF2 force field in AMBER18[5]. This dataset was down-sampled to 15,000 points by selecting evenly spaced points throughout the trajectory. Wavefunctions for each sample point were calculated by GAUSSIAN16[6] at the B3LYP/6-31+G(d,p) level of theory. Energy decomposition was then performed using the Interacting Quantum Atoms (IQA) energy partitioning implemented in AIMAll[7]. Generation of the data was performed using our in-house Python pipeline named ICHOR[8].

Uncertainty-enhanced stratified sampling (UESS) was employed to generate a training set and internal and external validation sets comprising 5,000, 750 and 1,500 points, respectively. A description of these sets is given in Section 3.1.2 of the main text. Models were trained in FEREBUS using both direct and transfer learning. In the implementation in FEREBUS, the training of the source model in the transfer learning process is controlled by a knowledge compression coefficient η, and a relaxation weight ζ. The knowledge compression coefficient is the proportion of the training set used to generate the source model. The hyperparameters obtained by training the source model are then used to guide the optimisation of hyperparameters for the whole training set, and the relaxation weight represents the proportion of the optimisation steps used to optimise the source model hyperparameters during training. In this section, we compare transfer-learnt models with η representing 0.1, 1 and 10 % of the training set and various ζ to a direct-learnt model, which serves as a reference. The accuracy of these models for reproducing molecular energies and charges across the 1,500-point external validation set is shown in Figure S3.1 together with the training times for each model and the root-mean-square-error (RMSE) in the atomic energies.

As highlighted in the main text, transfer-learnt models can be trained almost two orders of magnitude faster than direct learnt models, depending on the η and ζ parameters used to generate the models. However, the main text also shows that there is a trade-off between the computational cost and accuracy, with parameters describing smaller source models and fewer relaxation steps leading to faster training times but also higher errors relative to the directly-learnt model. For the monomer models this trade-off is more favourable, with a greater proportion of transfer-learnt models obtaining sub kJ mol^{-1} accuracy for the atomic energies while still showing a substantial reduction in training time. This is likely due to the lower dimensionality of the formamide monomer making the training process easier.

Many of the transfer-learnt models are capable of reproducing the molecular properties predicted by the direct-learnt model with sub 0.1-kJ mol^{-1} and sub 0.1-me accuracy. The transfer-learnt model trained using η = 0.1 and ζ = 0.01 was chosen for further calculations as this model was found to accurately reproduce the molecular properties from the direct-learnt model with a significant reduction in training time. Another candidate was the transfer learnt model with η = 0.1 and ζ = 0, but this was not selected to avoid potential issues with the source model hyperparameters not being optimised in the "frozen seed" model.

As in the main text, the cumulative error distributions were calculated for both the direct-learnt and transfer-learnt models. The error distributions in the atomic energies predicted by the direct-learnt and transfer-learnt models are compared in Figure S3.2. As the monomer model requires electrostatic interactions to be calculated during simulation of dimers, error distributions for the multipole moments up to the hexadecapole moment are also compared in Figures S3.3 to S3.27.

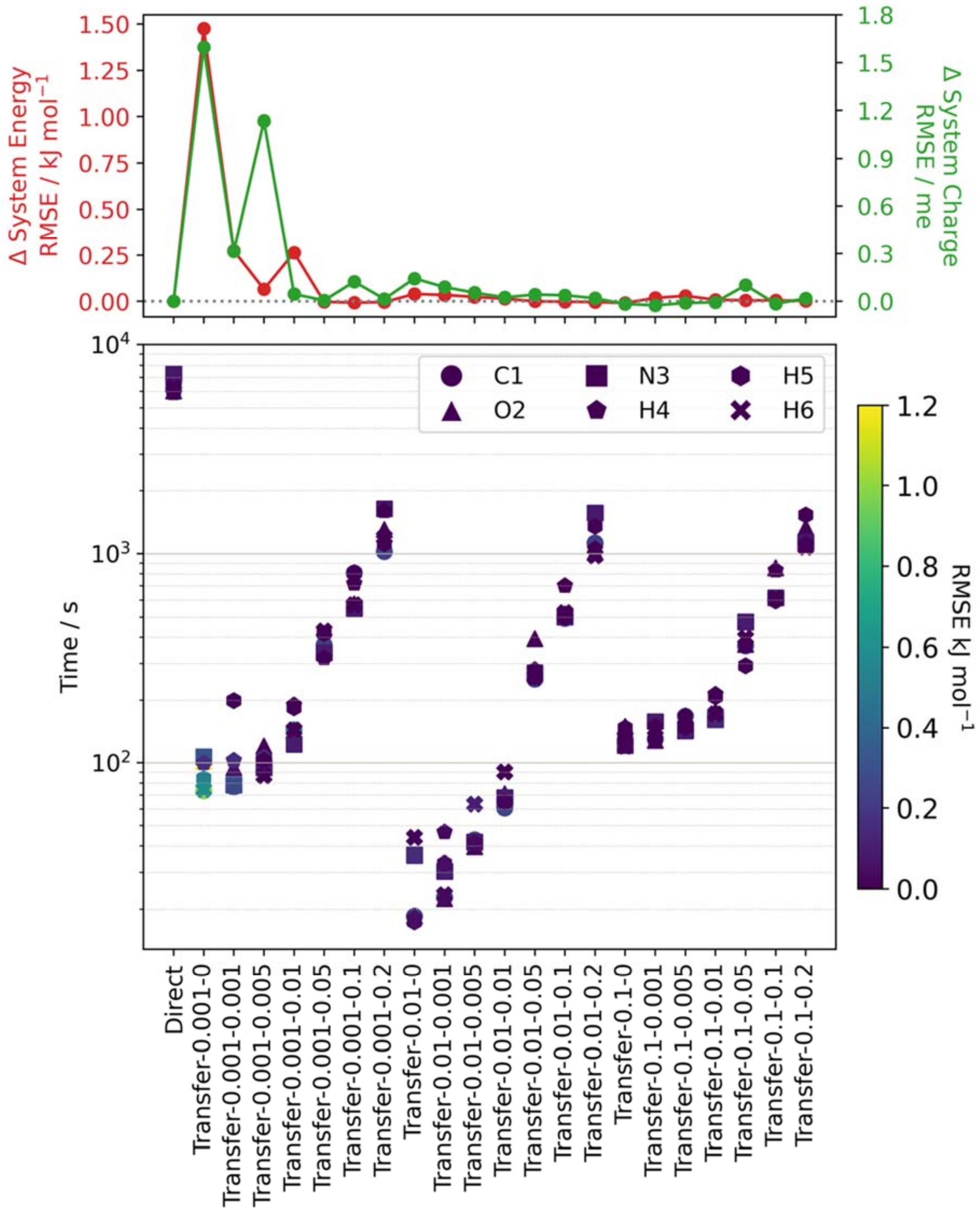

Figure S3.1. (Top) RMSE on the formamide monomer energy (red) and charge (green) from transfer-learnt models relative to a direct learnt model across a constant 1,500-point external validation set. (Bottom) Training times for individual atoms, using 20 cores of a single compute node comprising two Intel "Cascade Lake" Xeon Gold 6230 chips, compared to the RMSE in the atomic energies across the 1,500-point validation set. The labels indicate the knowledge compression coefficient, η, and relaxation weight, ζ, in the form "Transfer-η-ζ"exhausting the 3 × 7 = 21 possibilities.

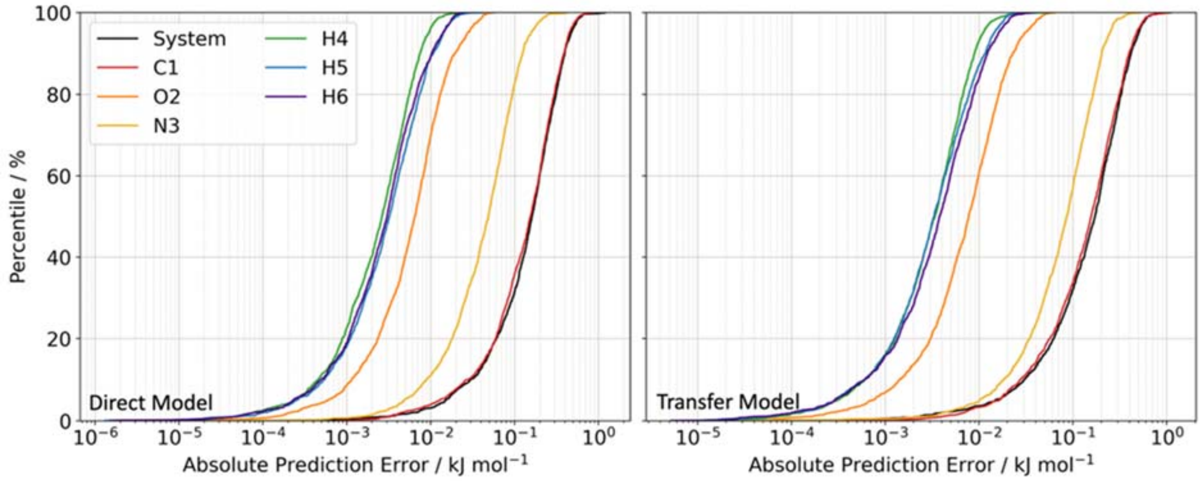

Figure S3.2. S-curves showing the absolute error in the IQA energies predicted by the direct and transfer-learnt formamide monomer models. Errors in the atomic energies are shown with coloured lines and the error in the total system energy (i.e. the whole monomer) is shown by the black line.

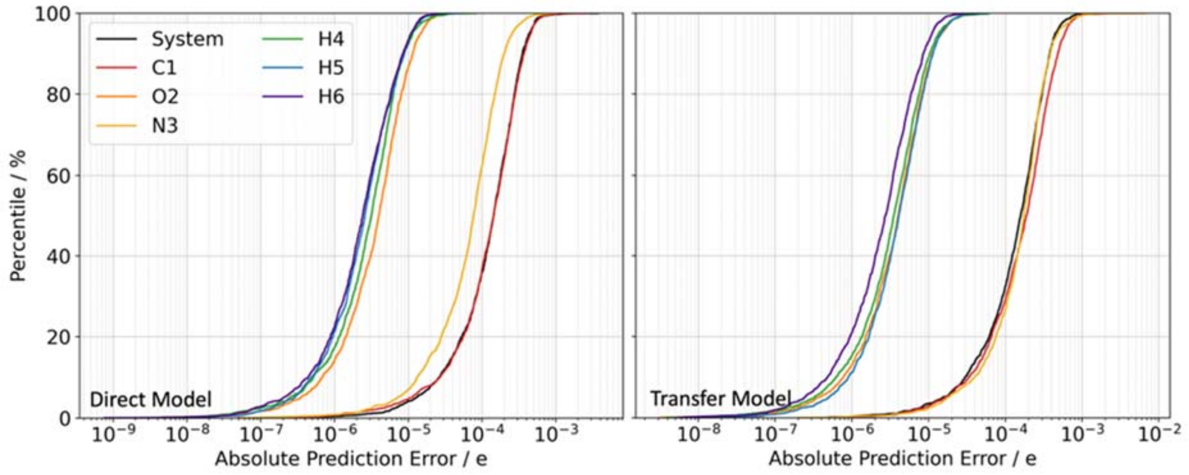

Figure S3.3. S-curves showing the absolute error in the atomic charges predicted by the direct and transfer-learnt formamide monomer models. Errors in the atomic charges are shown with coloured lines and the error in the total system charge (i.e. the whole monomer) is shown by the black line.

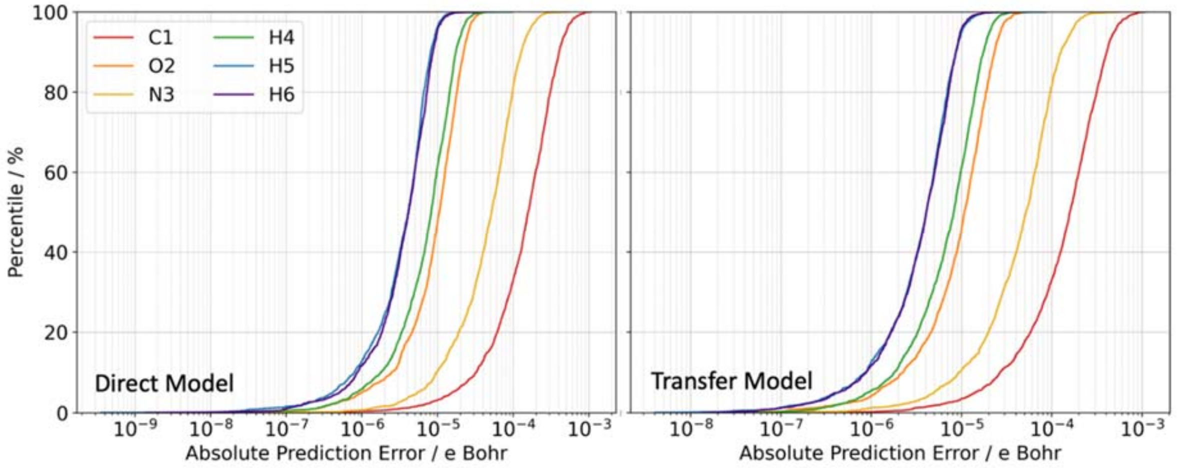

Figure S3.4. S-curves showing the absolute error in the predicted Q10 component of the atomic dipole moment from the direct and transfer-learnt formamide monomer models.

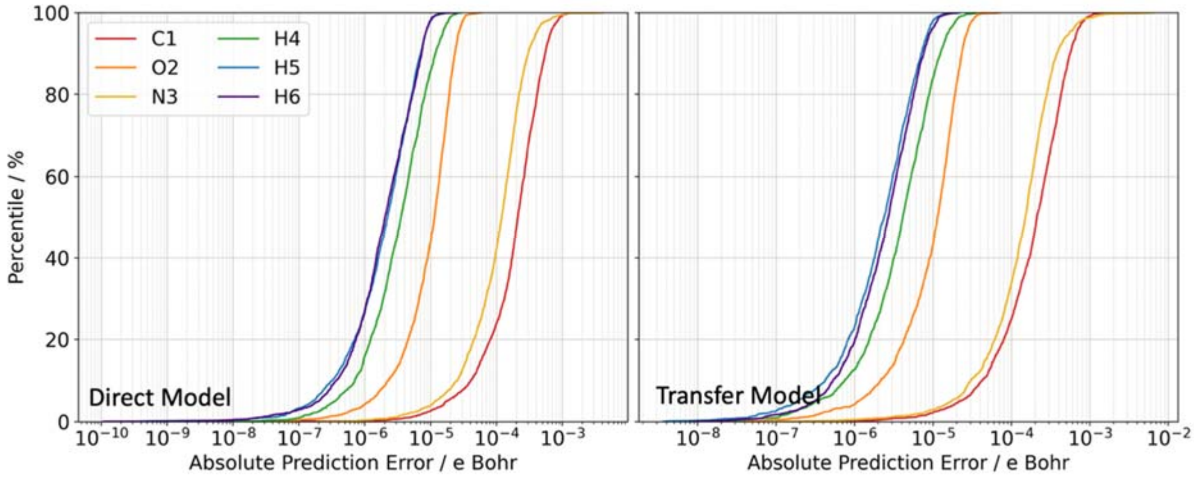

Figure S3.5. S-curves showing the absolute error in the predicted Q11c component of the atomic dipole moment from the direct and transfer-learnt formamide monomer models.

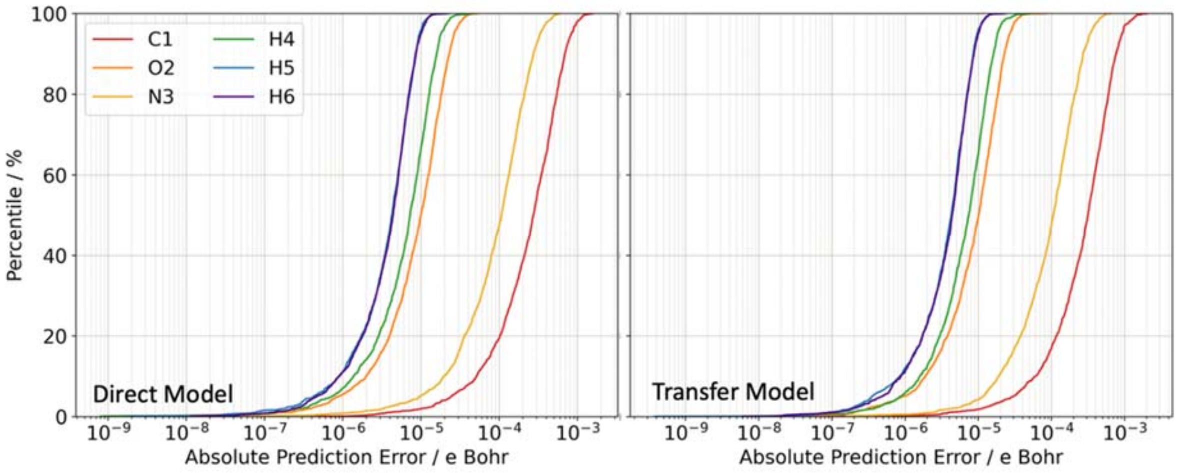

Figure S3.6. S-curves showing the absolute error in the predicted Q11s component of the atomic dipole moment from the direct and transfer-learnt formamide monomer models.

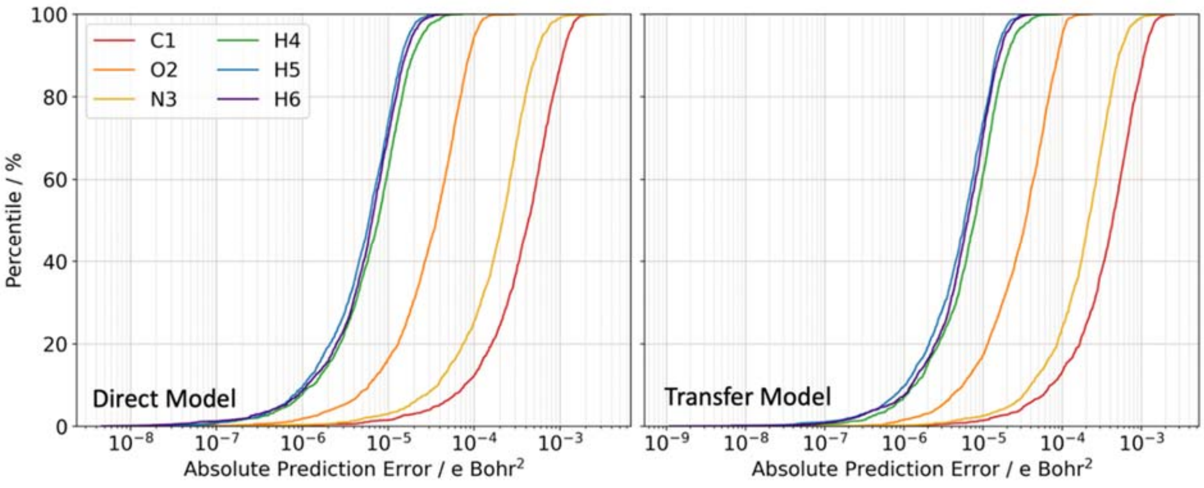

Figure S3.7. S-curves showing the absolute error in the predicted Q20 component of the atomic quadrupole moment from the direct and transfer-learnt formamide monomer models.

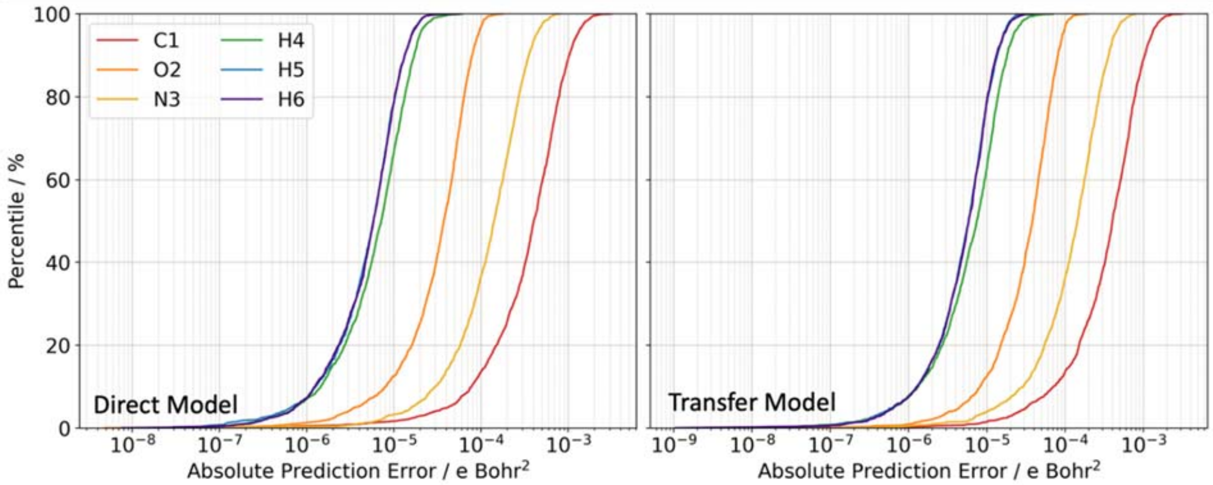

Figure S3.8. S-curves showing the absolute error in the predicted Q21c component of the atomic quadrupole moment from the direct and transfer learnt formamide monomer models.

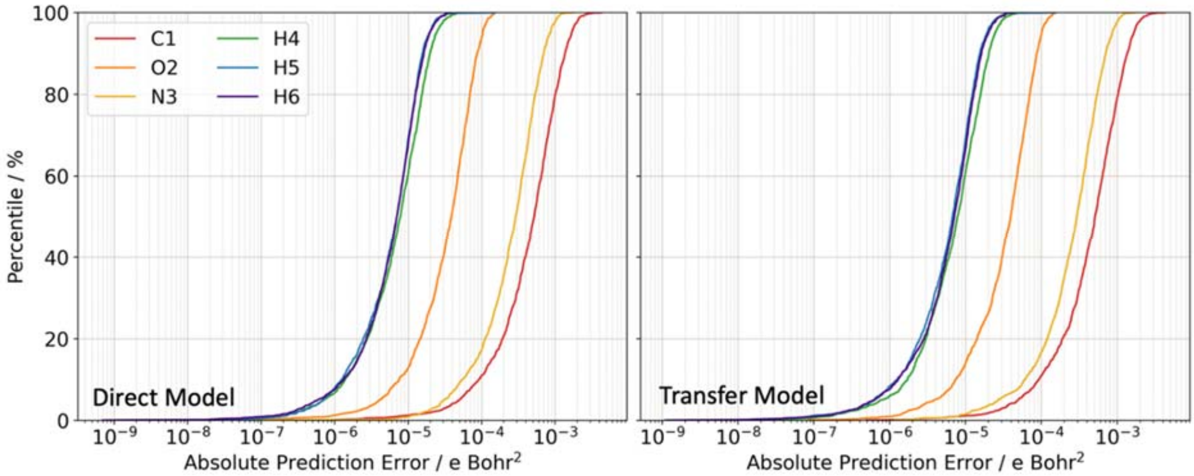

Figure S3.9. S-curves showing the absolute error in the predicted Q21s component of the atomic quadrupole moment from the direct and transfer-learnt formamide monomer models.

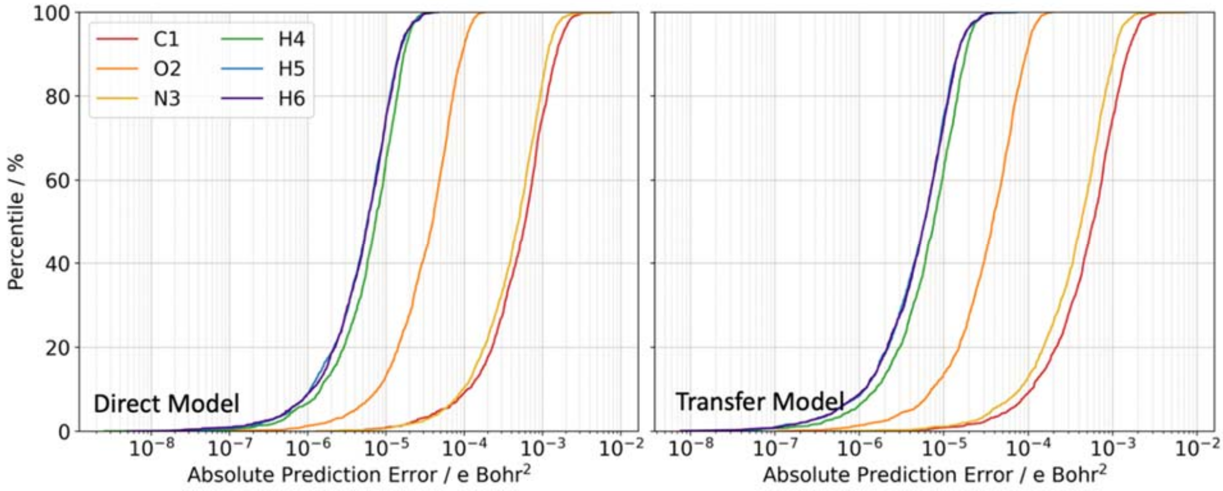

Figure S3.10. S-curves showing the absolute error in the predicted Q22c component of the atomic quadrupole moment from the direct and transfer-learnt formamide monomer models.

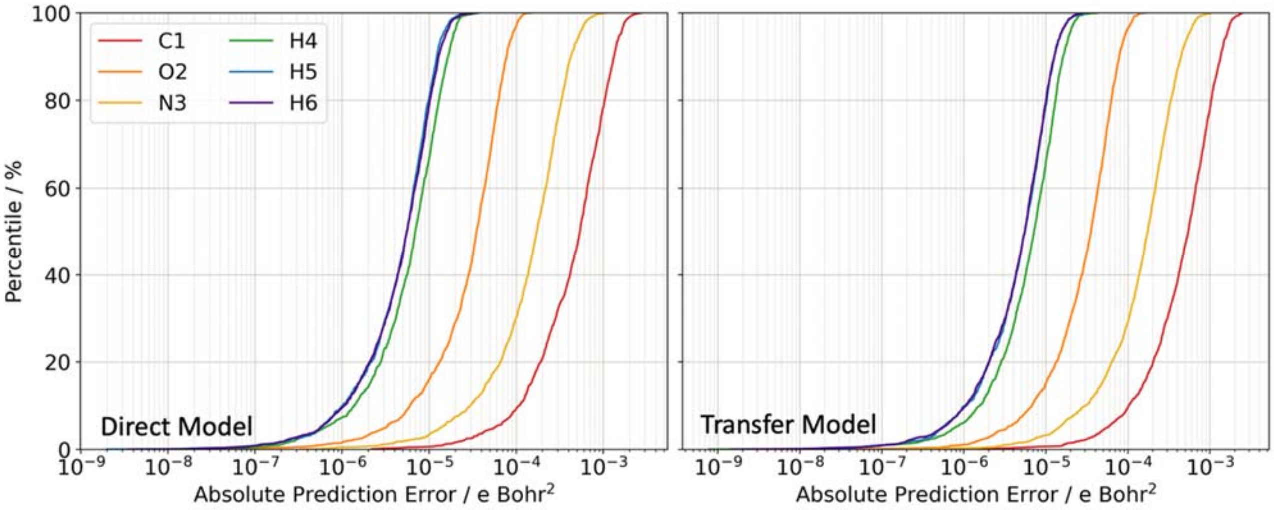

Figure S3.11 S-curves showing the absolute error in the predicted Q22s component of the atomic quadrupole moment from the direct and transfer learnt formamide monomer models.

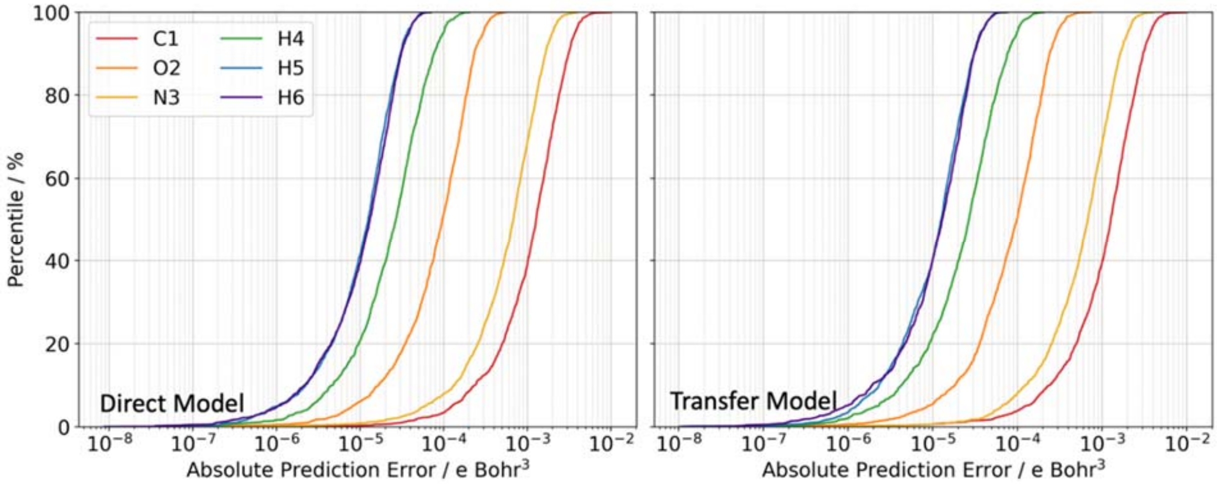

Figure S3.12. S-curves showing the absolute error in the predicted Q30 component of the atomic octupole moments from the direct and transfer-learnt formamide monomer models.

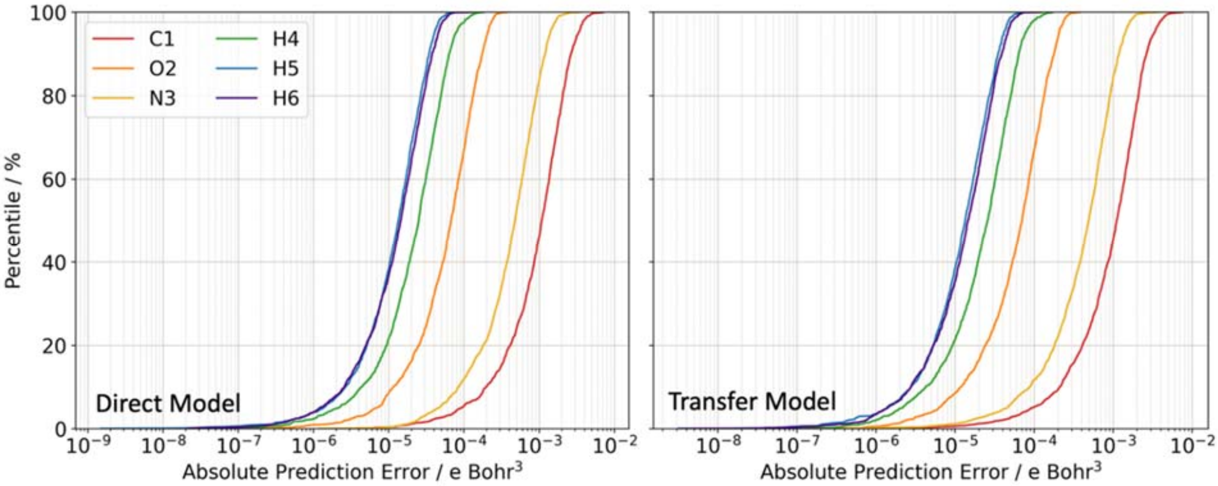

Figure S3.13. S-curves showing the absolute error in the predicted Q31c component of the atomic octupole moments from the direct and transfer-learnt formamide monomer models.

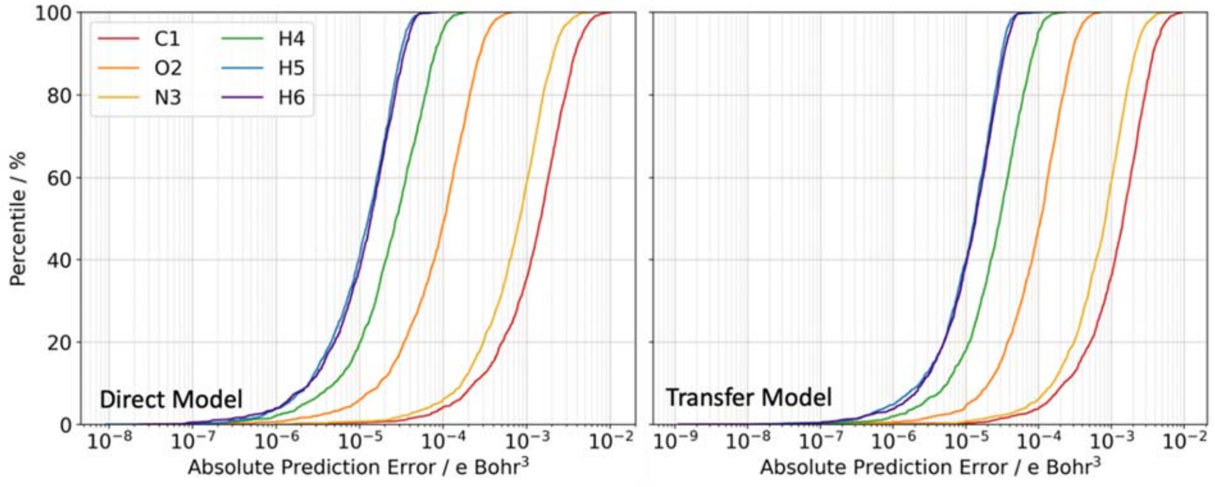

Figure S3.14. S-curves showing the absolute error in the predicted Q31s component of the atomic octupole moments from the direct and transfer-learnt formamide monomer models.

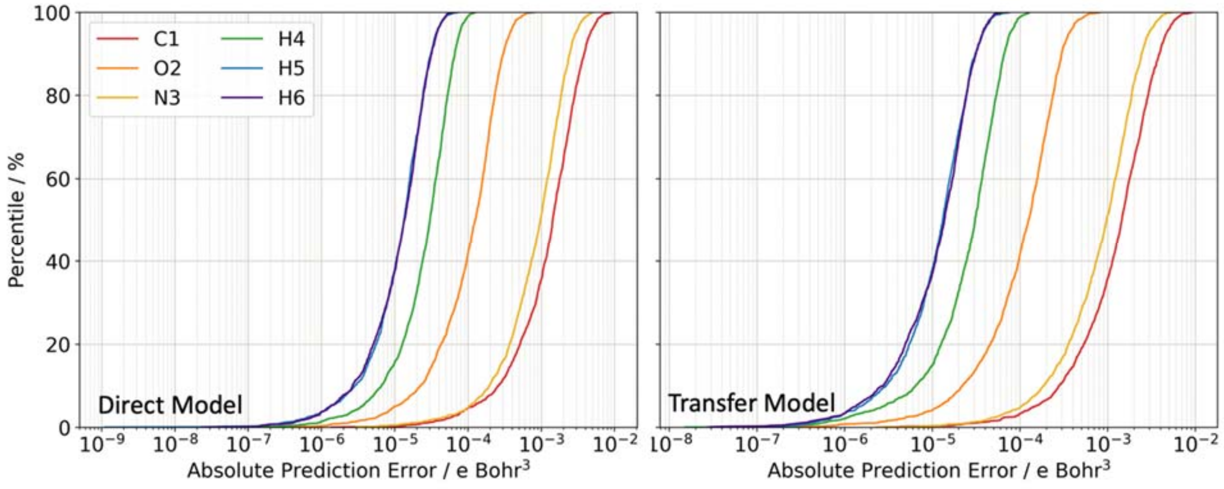

Figure S3.15. S-curves showing the absolute error in the predicted Q32c component of the atomic octupole moments from the direct and transfer learnt formamide monomer models.

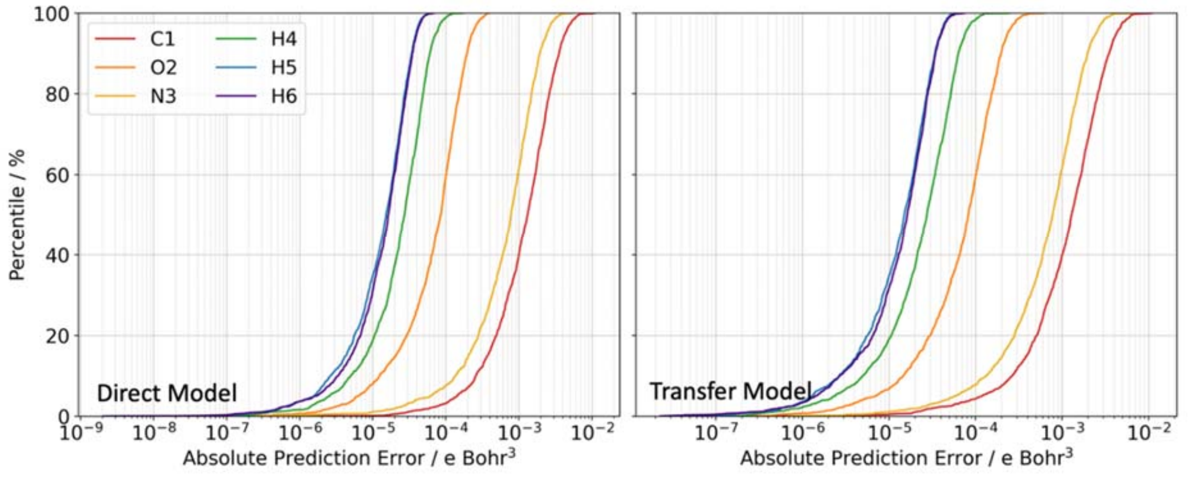

Figure S3.16. S-curves showing the absolute error in the predicted Q32s component of the atomic octupole moments from the direct and transfer learnt formamide monomer models.

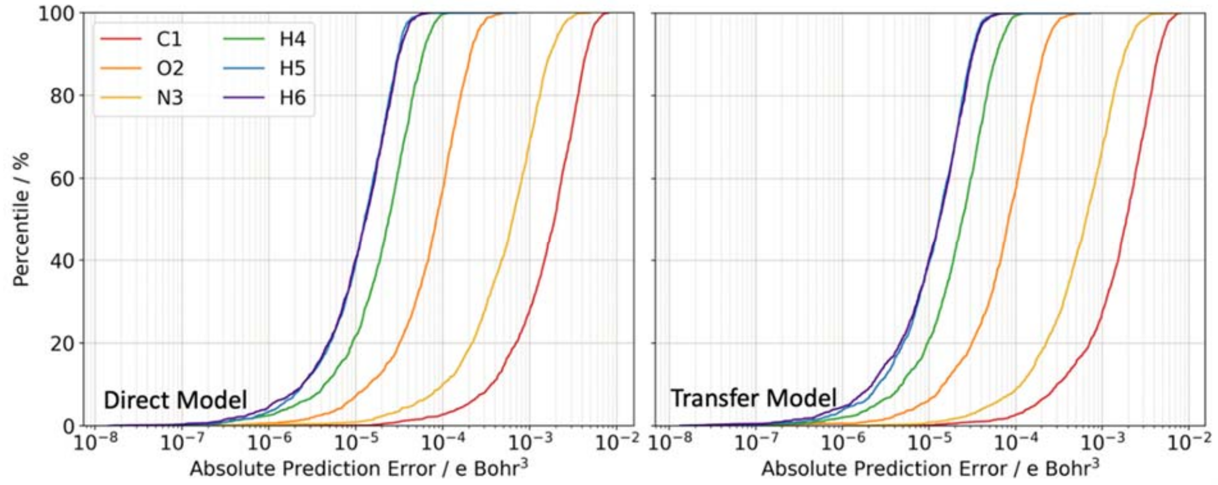

Figure S3.17. S-curves showing the absolute error in the predicted Q33c component of the atomic octupole moments from the direct and transfer learnt formamide monomer models.

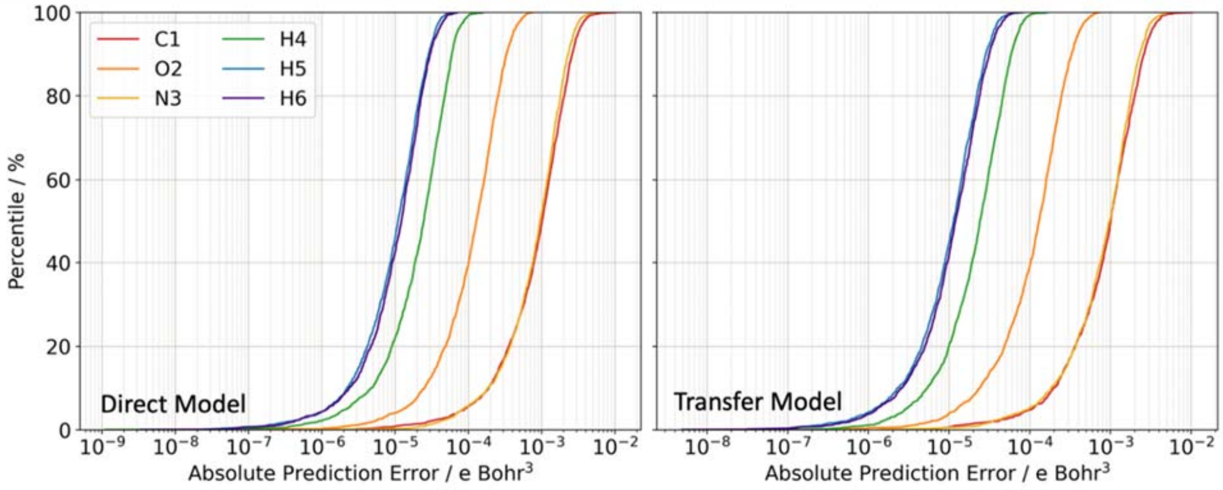

Figure S3.18. S-curves showing the absolute error in the predicted Q33s component of the atomic octupole moments from the direct and transfer-learnt formamide monomer models.

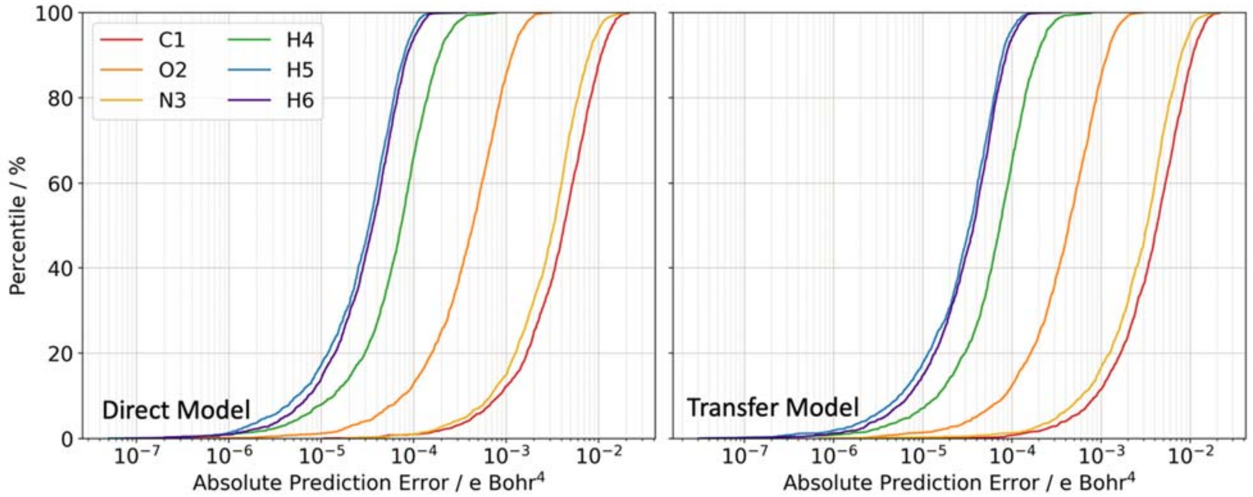

Figure S3.19. S-curves showing the absolute error in the predicted Q40 component of the atomic hexadecapole moment from the direct and transfer-learnt formamide monomer models.

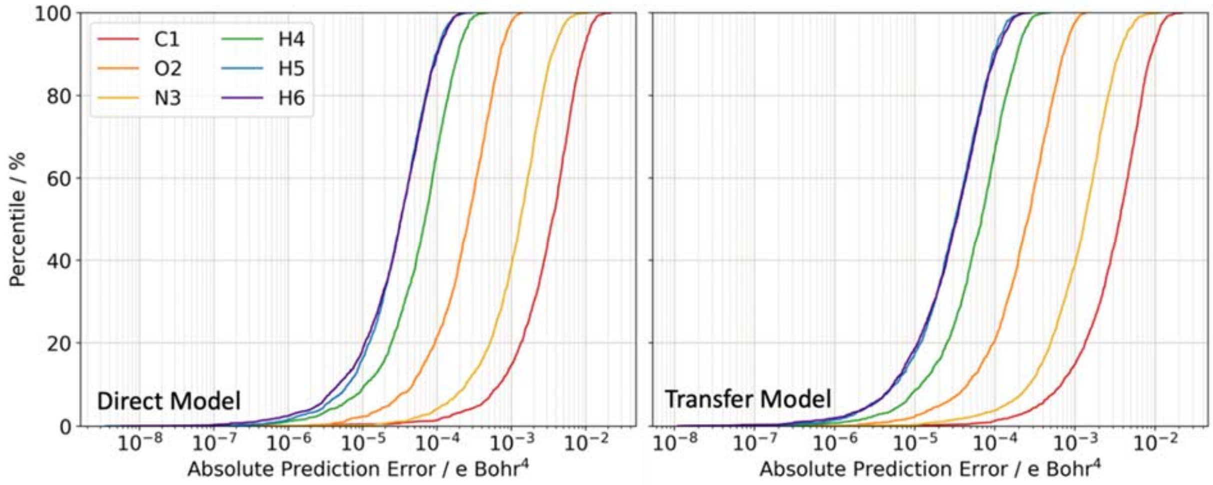

Figure S3.20. S-curves showing the absolute error in the predicted Q41c component of the atomic hexadecapole moment from the direct and transfer-learnt formamide monomer models.

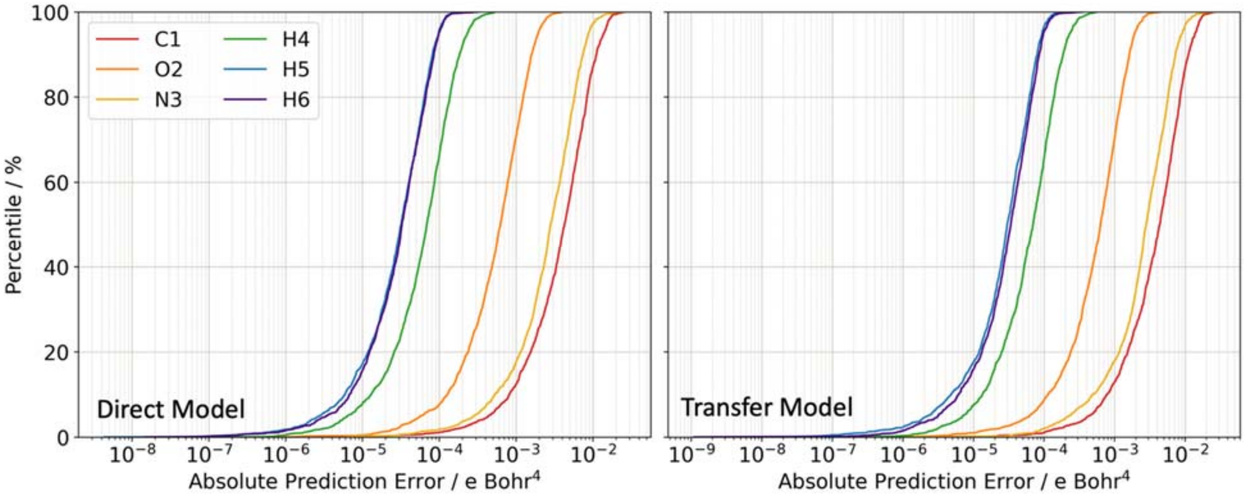

Figure S3.21. S-curves showing the absolute error in the predicted Q41s component of the atomic hexadecapole moment from the direct and transfer-learnt formamide monomer models.

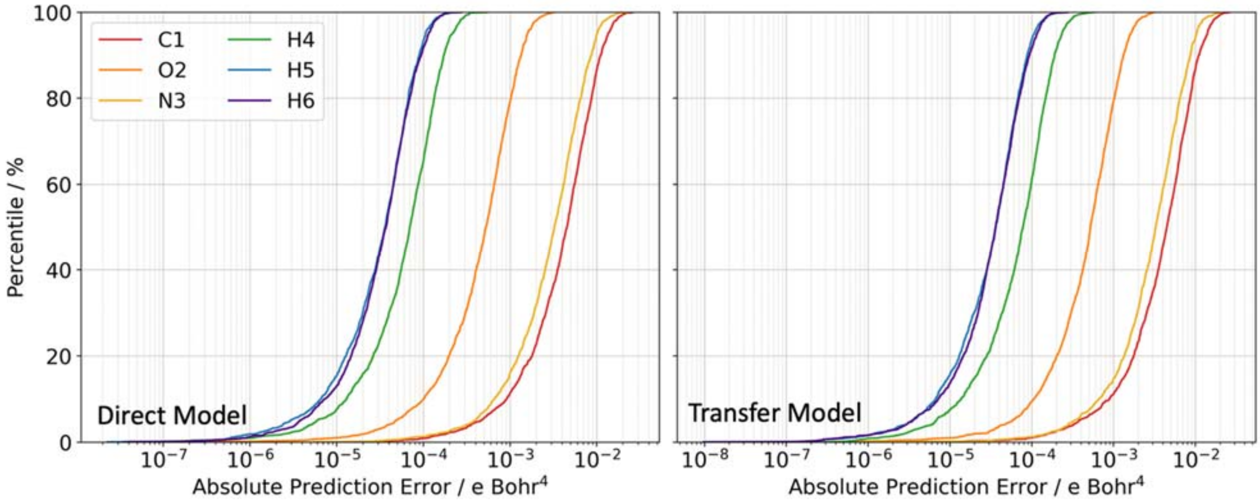

Figure S3.22. S-curves showing the absolute error in the predicted Q42c component of the atomic hexadecapole moment from the direct and transfer-learnt formamide monomer models.

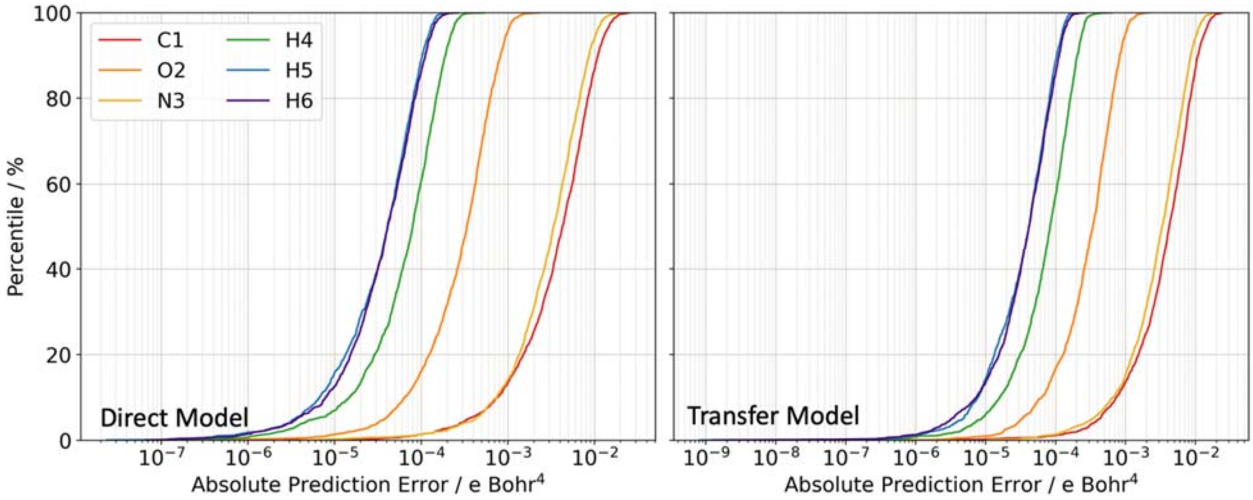

Figure S3.23. S-curves showing the absolute error in the predicted Q42s component of the atomic hexadecapole moment from the direct and transfer learnt formamide monomer models.

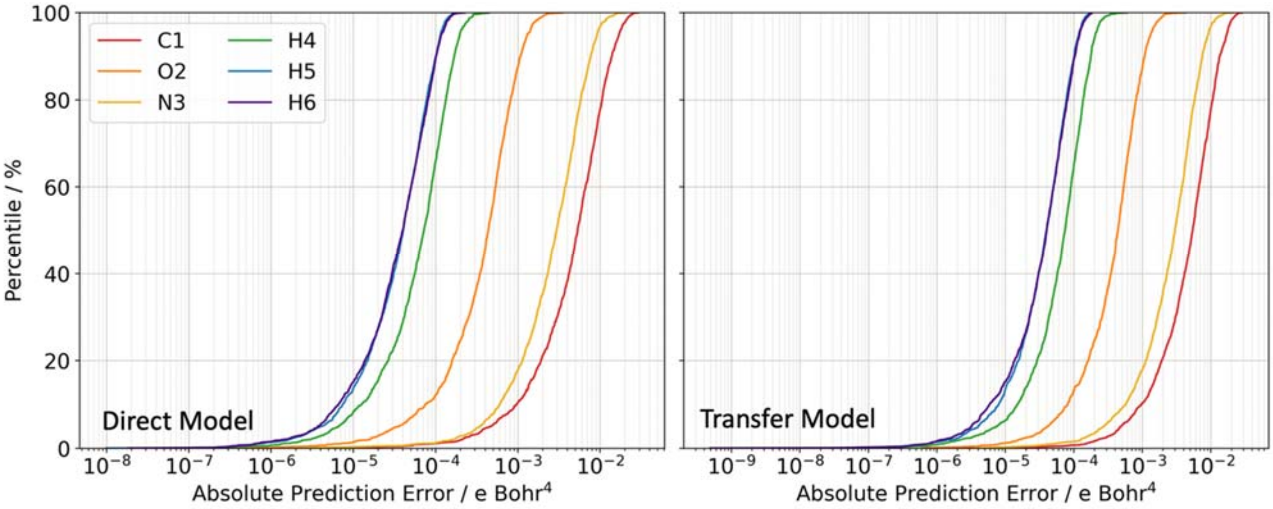

Figure S3.24. S-curves showing the absolute error in the predicted Q43c component of the atomic hexadecapole moment from the direct and transfer-learnt formamide monomer models.

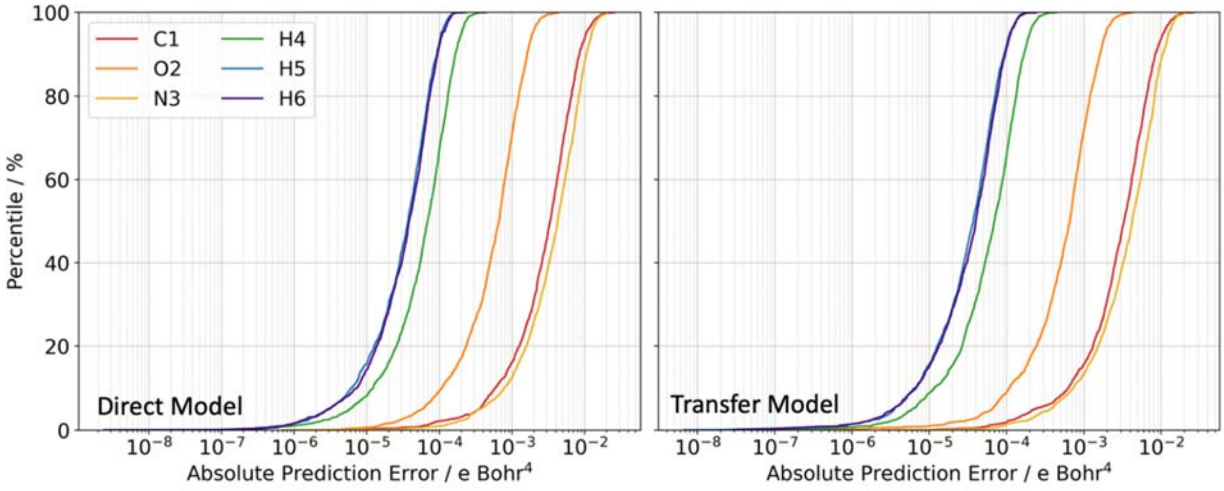

Figure S3.25. S-curves showing the absolute error in the predicted Q43s component of the atomic hexadecapole moment from the direct and transfer-learnt formamide monomer models.

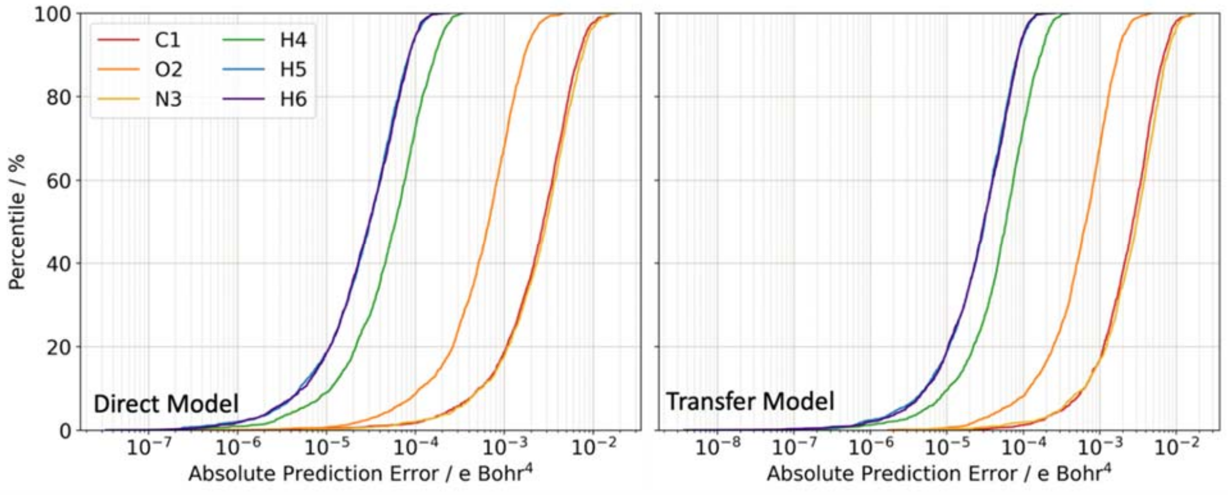

Figure S3.26. S-curves showing the absolute error in the predicted Q44c component of the atomic hexadecapole moment from the direct and transfer-learnt formamide monomer models.

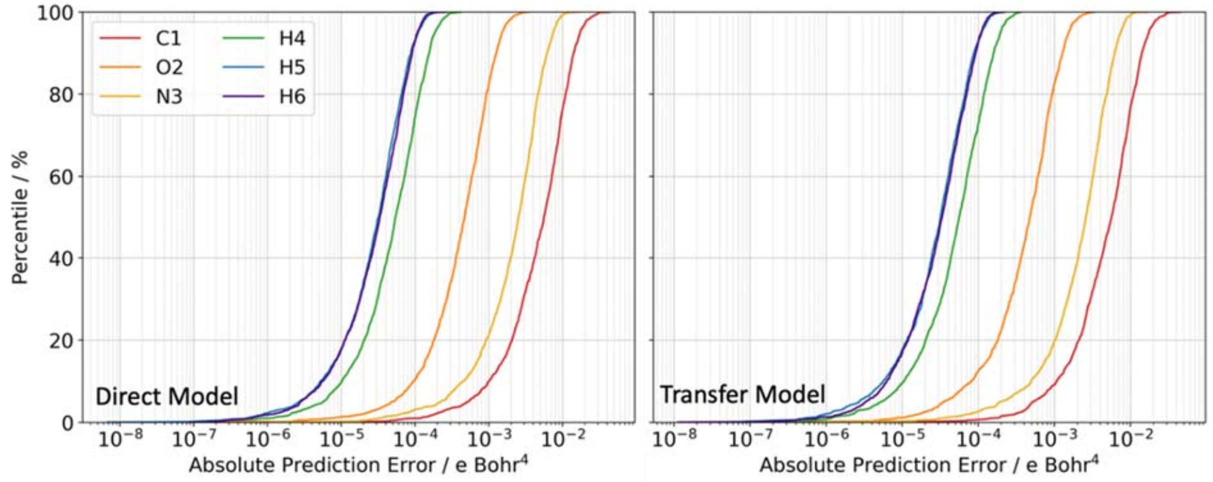

Figure S3.27. S-curves showing the absolute error in the predicted Q44s component of the atomic hexadecapole moment from the direct and transfer-learnt formamide monomer models.

A further test of the energy models is to perform geometry optimisations to see how well the optimum geometry is recovered by the FFLUX simulations. To do so, a series of twelve monomer geometries were generated by distorting the B3LYP/6-31+G(d,p) optimised monomer along the calculated normal mode coordinates obtained from GAUSSIAN16. Displacement steps were generated by scaling the normal mode coordinates by a factor of 0.15. The distorted geometries were then optimised as described in the main text. Because these optimisations are of a single molecule long-range electrostatic interactions are not required in these calculations. The root-mean-square deviation (RMSD) of each of the structures compared to the reference B3LYP/6-31+G(d,p) geometry, and of the average energy across the twelve optimised geometries was calculated and is presented in Table 1 in the main text, showing that both the direct and transfer-learnt GPR models recover the energy and geometry from the training level of theory well. It can therefore be concluded that transfer learning with appropriately chosen parameters can enable significant reductions in training time with a negligible effect on accuracy.

A further test to see how well the potential energy surface is recovered is to calculate the vibrational frequencies. Here we use the finite-difference method implemented in the Phonopy package[9] with FFLUX as the force calculator to obtain the normal mode coordinates and frequencies. As outlined in the main text, we used the FFLUX-optimised monomer for each of the models as the starting point for their respective frequency calculations. Table S3.1 compares the calculated frequencies to those calculated using the B3LYP/6-31+G(d,p) training level of theory.

Table S3.1. Vibrational frequencies (in cm^{-1}) of the formamide monomer calculated using the direct- and transfer-learnt monomer models. Absolute differences (Δ) from the reference vibrational frequencies calculated using B3LYP/6-31+G(d,p) are also given.

Mode	Assignment	B3LYP	FFLUX Direct	Δ	FFLUX Transfer	Δ
1	NH_2 wagging	256.79	235.2	21.59	243.39	13.40
2	NCO scissoring	565.43	563.56	1.87	568.74	3.31
3	NH_2 torsional twist	636.8	632.72	4.08	634.45	2.35
4	CH out-of-plane bend	1035.41	1034.48	0.93	1032.32	3.09
5	NH_2 rocking	1054.27	1072.72	18.45	1069.64	15.37
6	CN stretch	1270.31	1301.68	31.37	1294.26	23.95
7	OCH scissoring	1417.8	1417.68	0.12	1430.36	12.56
8	NH_2 scissoring	1621.48	1634.46	12.98	1637.77	16.29
9	C=O stretch	1797.05	1811.78	14.73	1809.55	12.50
10	CH Stretch	2976.79	3014.41	37.62	2981.35	4.56
11	Symmetric NH_2 stretch	3588.47	3595.21	6.74	3601.8	13.33
12	Asymmetric NH_2 Stretch	3733.06	3745.65	12.59	3728.94	4.12

The vibrational frequencies are recovered relatively well by the monomer models, with mean absolute errors respectively equating to 0.16 and 0.12 kJ mol^{-1} for the direct- and transfer-learnt models. The maximum errors in the vibrational frequencies also equate to energetic errors that are sub-kJ mol^{-1} and, therefore, firmly within the realm of chemical accuracy (approximately 4.2 kJ mol^{-1}). These values again show that direct and transfer learning can produce models of similar accuracy.

Infrared (IR) spectra were also calculated using the two models and are compared to the reference B3LYP/6-31+G(d,p) spectrum in Figure S3.28. The spectra were calculated as described in the main text, with the final spectrum obtained by averaging spectra from 25 MD runs.

Both models reproduce the training level of theory reasonably well, with good agreement in the relative intensities of the peaks between 1000 and 3000 cm^{-1} and with only two significant deviations outside of this range: (1) the NH_2 wagging at approximately 250 cm^{-1} is incorrectly predicted to have zero intensity, and (2) the (relative) intensities of the peaks associated with the symmetric and asymmetric NH stretches between 3500 and 4000 cm^{-1} are significantly overestimated.

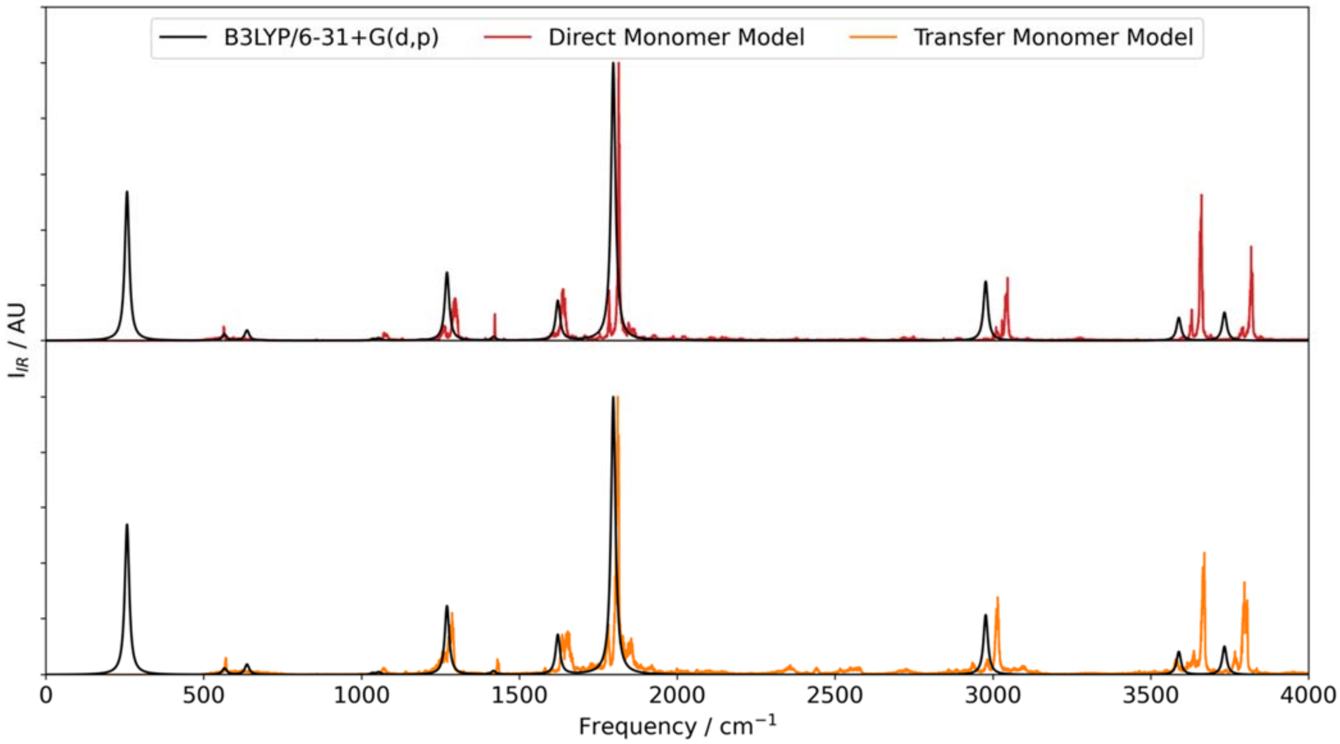

Figure S3.28. IR spectra calculated using the direct-learnt monomer model (red) and the transfer-learnt model (orange) compared to the B3LYP/6-31+G(d,p) spectrum (black).

The absence of the NH_2 wagging peak in the simulated spectra could be due either to inadequate sampling in the MD simulations or to errors in the calculated intensities due to the GPR model. To investigate further, we calculated the spectral density from the Fourier transform of the velocity autocorrelation function, $VAF(t)$, given by

$$VAF(t) = \frac{\langle \boldsymbol{v}_i(t_0) \cdot \boldsymbol{v}_i(t) \rangle}{\langle \boldsymbol{v}_i(t_0) \cdot \boldsymbol{v}_i(t_0) \rangle} \tag{S3.1}$$

where $\boldsymbol{v}_i(t)$ is the velocity of the i-th atom at time t and angular brackets indicate averages over atoms and the time origin t_0. The spectral density is shown in Figure S3.29.

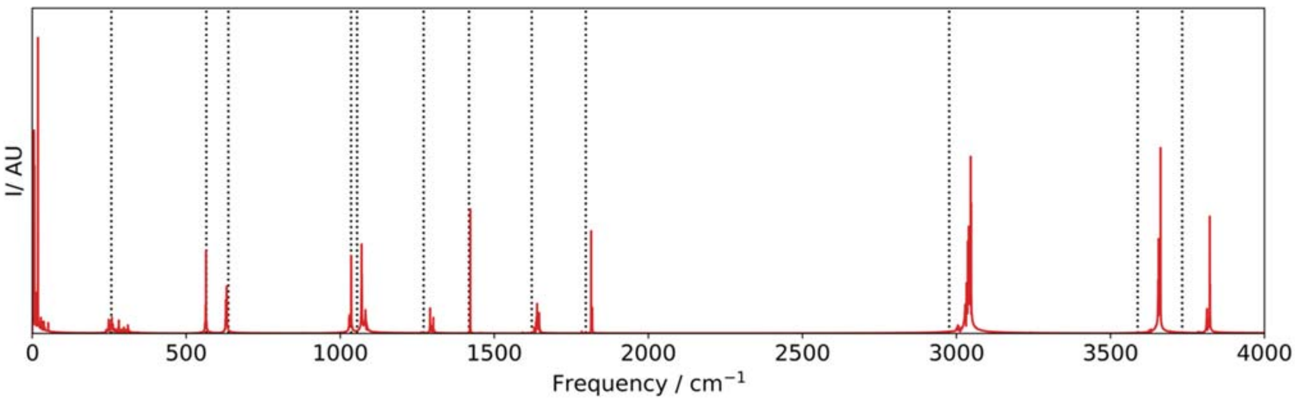

Figure S3.29. Spectral density of the formamide monomer calculated from the Fourier transform of the velocity autocorrelation function. The FFLUX spectrum (red) was averaged over five simulations. Black dotted lines indicate the harmonic frequencies obtained from a calculation at the B3LYP/6-31+G(d,p) training level of theory.

There is generally good agreement between features in the spectral density obtained from the FFLUX simulations and the frequencies predicted by B3LYP, albeit with notably larger errors in the higher-frequency modes. The spectral density shows a peak at approximately 250 cm^{-1} where the NH_2 wagging should be found in the IR spectrum. This indicates that the vibration is sampled in the simulations, and hence that the change in polarisation must be poorly captured by the GPR model. We note that, despite removing the average velocity autocorrelation function before applying the Fourier transform, the spectrum retains a large component centred around ω=0 cm^{-1}. Since no vibrations with such low frequencies were found in the finite-difference calculations, we attribute these to drift. The fact that this features does not appear in the IR spectrum derived from the total system dipole moment autocorrelation function is consistent with it being associated with translational or rotational vibrational modes.

There is generally good agreement between the FFLUX vibrational frequencies calculated using the finite-difference method and the MD method, with the largest differences for the CH and NH stretches where MD predicts higher frequencies than the finite-difference method (although the differences are less than 100 cm^{-1}). The frequencies of the stretches between 3000 and 4000 cm^{-1} are also slightly higher than obtained with the training level of theory. Given that the FFLUX finite-difference and B3LYP frequencies are both calculated within the harmonic approximation, the higher frequencies predicted by the MD method may be due to partial inclusion of anharmonic shifts in the MD simulations, despite the low temperature of 50 K at which the MD simulations were performed. This is examined in Section 7 of the SI.

4 Assignment of the Formamide Dimer Vibrational Modes

Table S4.1. Assignment of the normal modes of the formamide dimer obtained at the B3LYP/6-31+G(d,p) level of theory.

Mode	Frequency / cm^{-1}	Assignment
1	63.61	Intermolecular twist
2	137.39	Intermolecular wag (in-phase)
3	145.83	Intermolecular rocking
4	171.55	O···H stretch (in-phase)
5	178.90	Intermolecular wag (out-of-phase)
6	215.04	O···H stretch (out-of-phase)
7	492.73	NH2 wag (out-of-phase)
8	503.43	NH2 wag (in-phase)
9	609.42	NCO scissoring (in-phase)
10	631.35	NCO scissoring (out-of-phase)
11	825.54	NH2 torsional twist (out-of-phase)
12	864.14	NH2 torsional twist (in-phase)
13	1049.23	CH out of plane bend (out-of-phase)
14	1058.91	CH out of plane bend (in-phase)
15	1096.75	NH2 rocking (out-of-phase)
16	1103.66	NH2 rocking (in-phase)
17	1334.23	CN stretch (out-of-phase)
18	1347.53	CN stretch (in-phase)
19	1422.00	OCH scissoring (in-phase)
20	1422.24	OCH scissoring (out-of-phase)
21	1644.50	NH2 scissoring (in-phase)
22	1651.65	NH2 scissoring (out-of-phase)
23	1750.48	C=O stretch (in-phase)
24	1780.53	C=O stretch (out-of-phase)
25	2999.16	CH stretch (out-of-phase)
26	3002.21	CH stretch (in-phase)
27	3293.57	Symmetric NH2 stretch (in-phase)
28	3338.99	Symmetric NH2 stretch (out-of-phase)
29	3683.01	Asymmetric NH2 stretch (in-phase)
30	3683.49	Asymmetric NH2 stretch (out-of-phase)

5 Transferability of Monomer Multipole Moments

To assess the transferability of multipole moments predicted by the formamide monomer model to the dimer system, a comparison of the 36 intermolecular atom-atom electrostatic energies in the dimer was performed.

The formamide dimer was optimised at the training level of theory and its wavefunction calculated. The wavefunction was then used for an IQA analysis in AIMAll, yielding the electrostatic energies V_{cl}^{AB} (see eqns 2-5 in the main text).

Each of the constituent monomers were than taken and the wavefunctions calculated and analysed using IQA scheme to obtain the atomic multipole moments. The multipole moments were then used to calculate the intermolecular atom-atom electrostatic energies at different electrostatic ranks, referred to by the quantity L', for the dimer. (L' denotes the highest multipolar rank present in a simulation and is described in more detail in the main text.)

The error in the energy of each interaction was then calculated as:

$$E = \left|V_{L'}^{AB} - V_{cl}^{AB}\right| \qquad (S5.1)$$

where V_{cl}^{AB} is the "true" electrostatic energy between atoms A and B from the IQA partitioning of the dimer, and $V_{L'}^{AB}$ is the electrostatic energy calculated from the multipole moments of the atoms in the constituent monomers at a given L'. These errors are presented as heatmaps in Figure S5.1.

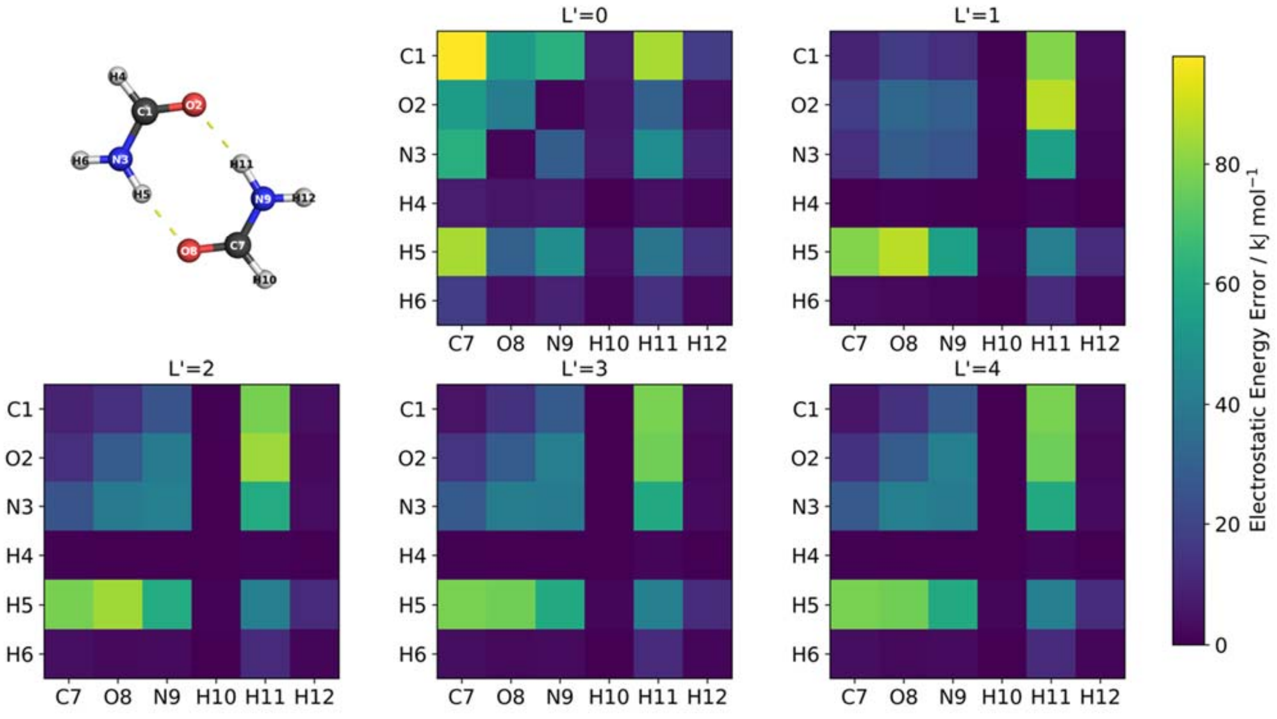

Figure S5.1. Heatmaps showing the errors in the intermolecular atom-atom electrostatic energies calculated using monomeric multipole moments compared to the "true" electrostatic energies from an IQA partitioning of the dimer.

The heatmaps show that as the multipolar rank is increased the monomeric moments are better able to represent the V_{cl}^{AB} of the dimer. The errors in the electrostatic energies from low-order moments indicate that these are not transferable between the monomer to the dimer but, as the multipolar rank increases, there is some cancellation of errors. For example, at $L' = 0$ the C-C interaction exhibits an error of over 90 kJ mol^{-1}. However, adding dipoles at $L' = 1$ reduces this error to below 20 kJ mol^{-1}. But then again, at the maximum rank of $L' = 4$, the errors in the electrostatic interactions are still large, with a mean absolute error of 21.1 kJ mol^{-1} across the 36 interactions. The largest errors are associated with the hydrogen atoms participating in the hydrogen bonding, which is due to the multipole moments in the monomer not accounting for the intermolecular polarisation in the dimer.

From this analysis, we conclude that the monomeric multipole moments are not transferable to the dimer, but electrostatic interactions with $L' = 4$ (multipole moments up to the hexadecapole moment) nevertheless offer a reasonable representation of the "true" dimer electrostatic energies.

6 Optimisation of Lennard-Jones Parameters for the Monomer Model

The monomeric Gaussian process regression (GPR) models used in FFLUX simulations are able to predict atomic multipole moments, which allows the electrostatic interactions between molecules in a dimer (or other multi-molecule system) to be predicted. However, the monomeric models are unable to predict dispersive and repulsive intermolecular interactions, nor a suitable non-bonded potential. There are several different potentials available in DL_POLY, and hence in DL_FFLUX. In this work a 12-6 Lennard-Jones potential was used, which has the following functional form:

$$U(r_{ij}) = \frac{A_{ij}}{r_{ij}^{12}} - \frac{B_{ij}}{r_{ij}^{6}} \tag{S6.1}$$

where A_{ij} and B_{ij} are parameters for the interactions between atoms i and j. We initially (i.e. before optimisation) used the A parameters listed in Table S6.1, which were derived in our previous work on formamide[10]. Only the A parameters, representing repulsive interactions, were used for a fair comparison to the dimer model, where all information comes from a B3LYP wavefunction that formally contains no measure of dispersion.

Table S6.1. Initial non-bonded parameters for formamide calculations using FFLUX. Only the A parameter, representing the repulsive interactions between atoms, was optimised, as the B parameter was set to zero in the calculations presented in this work.

Atom	A / kJ mol^{-1} Å^{12}	B / kJ mol^{-1} Å^{6}
C	13,534,048	12,606.560
N	10,891,864	11,486.320
O	3,440,600	3900.368

As described in the SI of Reference 10 the following mixing rules were used to obtain parameters for the interactions between pairs of atoms of different types:

$$A_{ij} = \sqrt{A_{ii}A_{jj}} \tag{S6.2}$$

$$B_{ij} = \sqrt{B_{ii}B_{jj}} \tag{S6.3}$$

Only the initial A parameters in Table S4.1 were optimised for use in $L' = 4$ simulations in which monopole, dipole, quadrupole, octupole and hexadecapole moments are used to describe the intermolecular electrostatic interactions. The parameters were adapted by scaling the A values by a factor n to obtain scaled parameters A_{ij}^{*} according to:

$$A_{ij}^{*} = nA_{ij} \tag{S6.4}$$

The parameters were scaled from 70 % to 130 % (i.e. from 0.70 to 1.30) of the initial values in steps of 2.5 % (i.e. 0.025), and the formamide dimer was optimised with each parameter set as described in the main text. The parameter set that minimised the root-mean-square-deviation (RMSD) of the optimised dimer compared to the B3LYP/6-31+G(d,p) geometry was then selected. While not necessarily the best way of obtaining non-bonded parameters, this does highlight the point that non-bonded potentials such as those for a Lennard-Jones potential can in principle be adjusted to obtain a desired result.

The RMSDs for each parameter set are given in Table S6.2. The best performing set was found to be the initial parameters scaled by 0.775 (*i.e.* decreased by 22.5 %), which is highlighted in bold red text in the table. These parameters differ from those derived in our previous work for two reasons: (i) in this work the dispersion parameter has been set to zero, effectively resulting in a different functional form, and (ii) the parameters in this work are derived for $L' = 4$, which is a higher electrostatic rank than was used in our previous work ($L' = 3$).

Table S6.2. RMSD of the formamide dimer obtained from FFLUX geometry optimisations with the monomer model and scaled Lennard-Jones parameters compared to the B3LYP/6-31+G(d,p) optimised geometry.

Scaling n	RMSD / Å	Scaling n	RMSD / Å
0.700	0.065	1.025	0.095
0.725	0.059	1.050	0.101
0.750	0.055	1.075	0.107
**0.775**	**0.053**	1.100	0.113
0.800	0.053	1.125	0.117
0.825	0.055	1.150	0.124
0.850	0.058	1.175	0.129
0.875	0.062	1.200	0.134
0.900	0.067	1.225	0.139
0.925	0.072	1.250	0.144
0.950	0.078	1.275	0.149
0.975	0.083	1.300	0.154
1.000	0.090		

7 Anharmonic Infrared Spectra

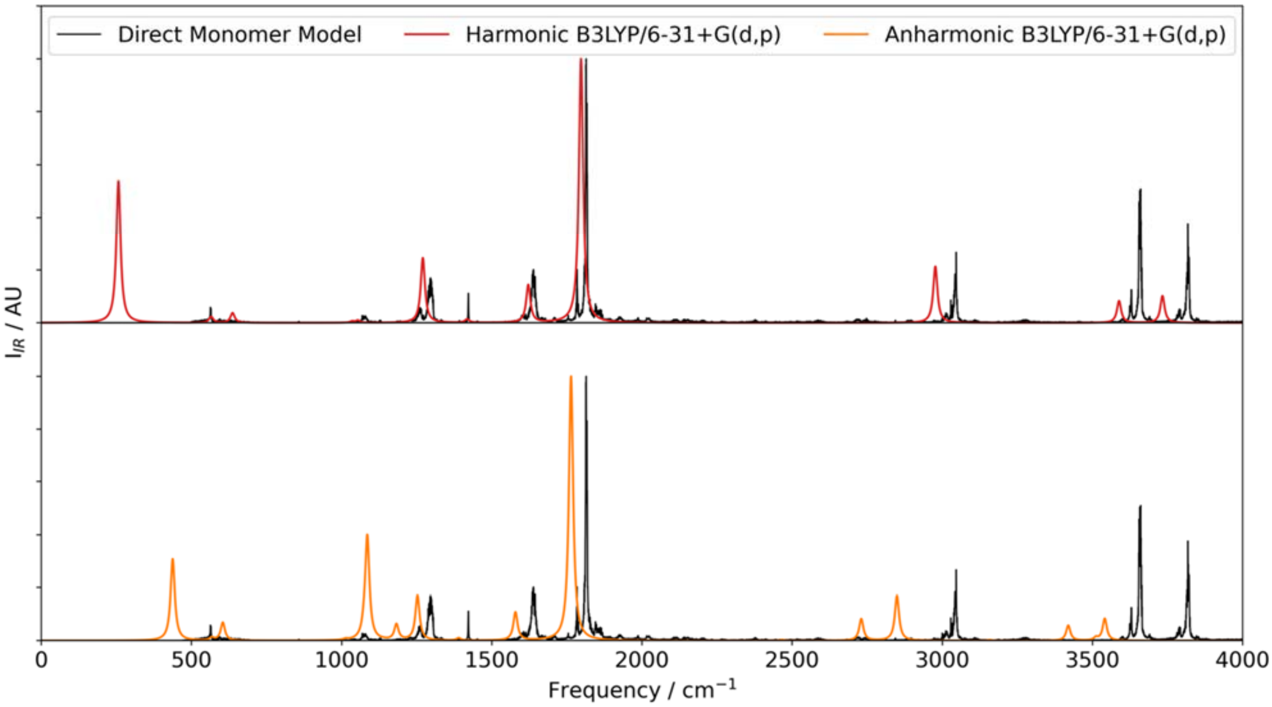

Figure S7.1. Harmonic (red) and anharmonic (orange) IR spectra of the formamide monomer calculated at the training B3LYP/6-31+G(d,p) level of theory and compared to the spectrum obtained from MD simulations in FFLUX with the direct-learnt monomer model (black). The low temperature used in the MD simulations results in anharmonic effects not being captured in the FFLUX spectrum.

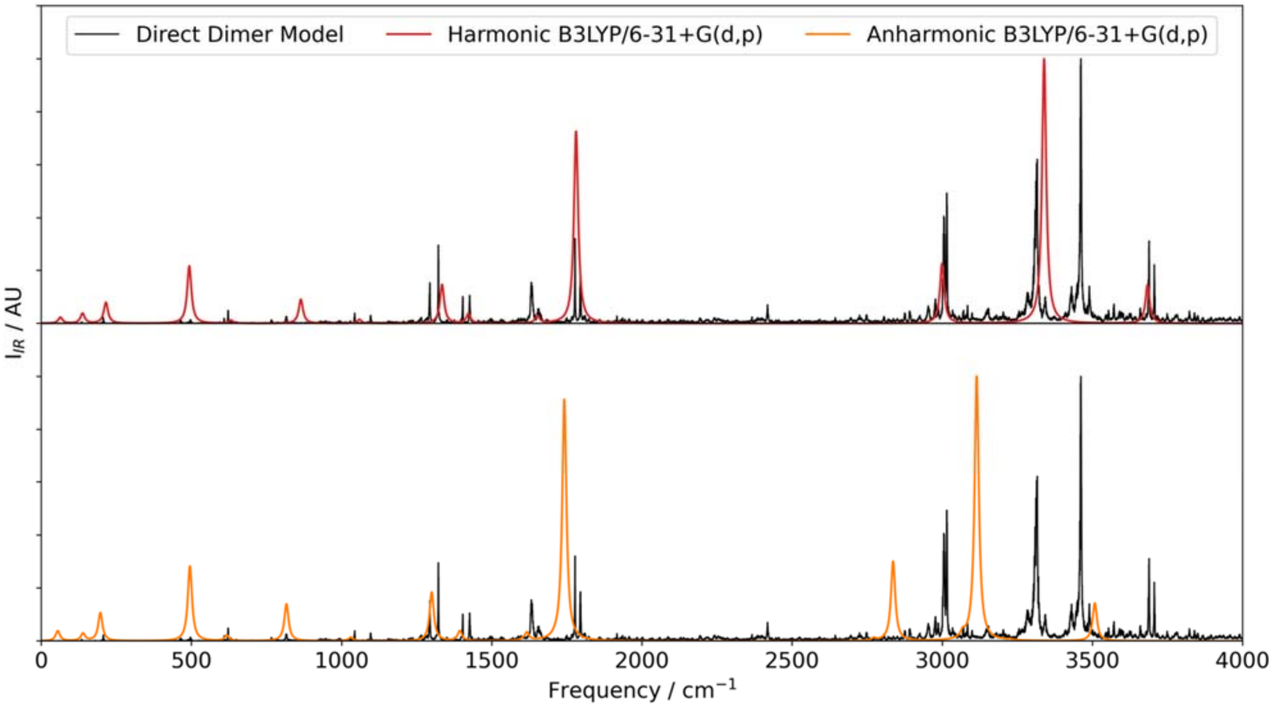

Figure S7.2. Harmonic (red) and anharmonic (orange) IR spectra of the formamide dimer calculated at the training B3LYP/6-31+G(d,p) level of theory and compared to the spectrum obtained from MD simulations in FFLUX with the direct learnt dimer model (black). The low temperature of the MD simulations results in anharmonic effects not being captured in the FFLUX spectrum.

References

(1) Di Pasquale, N.; Bane, M.; Davie , S. J.; Popelier, P. L. A. FEREBUS: Highly Parallelized Engine for Kriging Training. *J. Comput. Chem.* **2016**, *37*, 2606-2616.

(2) Burn, M. J.; Popelier, P. L. A. FEREBUS: a High-performance Modern Gaussian Process Regression Engine. *Digital Discovery* **2023**, *2*, 152-164.

(3) Isamura, B. K.; Popelier, P. L. A. Metaheuristic optimisation of Gaussian process regression model hyperparameters: Insights from FEREBUS. *Artificial Intelligence Chemistry* **2023**, *1* (2), 100021.

(4) Yu, H.; Kim, S. Passive Sampling for Regression. In *2010 IEEE International Conference on Data Mining*, 2010; pp 1151-1156.

(5) D.A. Case; I.Y. Ben-Shalom; S.R. Brozell; D.S. Cerutti; T.E. Cheatham III; V.W.D. Cruzeiro; T.A. Darden; R.E. Duke; D. Ghoreishi; M.K. Gilson; et al. *AMBER 2018, University of California, San Francisco.* **2018**.

(6) M. J. Frisch; G. W. Trucks, H. B. S., G. E. Scuseria, M. A. Robb, J. R. Cheeseman, G. Scalmani, V. Barone, G. A. Petersson, H. Nakatsuji, X. Li, M. Caricato, A. V. Marenich, J. Bloino, B. G. Janesko, R. Gomperts, B. Mennucci, H. P. Hratchian, J. V. Ortiz, A. F. Izmaylov, J. L. Sonnenberg, Williams, F. Ding, F. Lipparini, F. Egidi, J. Goings, B. Peng, A. Petrone, T. Henderson, D. Ranasinghe, V. G. Zakrzewski, J. Gao, N. Rega, G. Zheng, W. Liang, M. Hada, M. Ehara, K. Toyota, R. Fukuda, J. Hasegawa, M. Ishida, T. Nakajima, Y. Honda, O. Kitao, H. Nakai, T. Vreven, K. Throssell, J. A. Montgomery Jr., J. E. Peralta, F. Ogliaro, M. J. Bearpark, J. J. Heyd, E. N. Brothers, K. N. Kudin, V. N. Staroverov, T. A. Keith, R. Kobayashi, J. Normand, K. Raghavachari, A. P. Rendell, J. C. Burant, S. S. Iyengar, J. Tomasi, M. Cossi, J. M. Millam, M. Klene, C. Adamo, R. Cammi, J. W. Ochterski, R. L. Martin, K. Morokuma, O. Farkas, J. B. Foresman and D. J. Fox. GAUSSIAN16. *GAUSSIAN16* **2016**.

(7) T. Keith, AIMAll Version 19, Gristmill Software, Overland Park, Kansas, USA, (aim.tkgristmill.com): 2019 (accessed 24/03/2024).

(8) Burn, M. J.; Popelier, P. L. A. ICHOR: A Modern Pipeline for Producing Gaussian Process Regression Models for Atomistic Simulations. *Materials Advances* **2022**, *3*, 8729-8739.

(9) Togo, A.; Tanaka, I. First Principles Phonon Calculations in Materials Science. *Scr. Mater.* **2015**, *108*, 1-5.

(10) Brown, M. L.; Skelton, J. M.; Popelier, P. L. A. Construction of a Gaussian Process Regression Model of Formamide for Use in Molecular Simulations. *J. Phys. Chem. A* **2023**, *127* (7), 1702-1714.
